# Control of cerium oxidation state through metal complex secondary structures†
†Electronic supplementary information (ESI) available: NMR spectra, UV-Vis spectra, FTIR spectra, Evans' method data, field dependence data, XAS spectra, electrochemical data, DFT coordinates and rendered molecular orbitals. CCDC 1404761–1404763. For ESI and crystallographic data in CIF or other electronic format see DOI: 10.1039/c5sc02607e


**DOI:** 10.1039/c5sc02607e

**Published:** 2015-08-11

**Authors:** Jessica R. Levin, Walter L. Dorfner, Patrick J. Carroll, Eric J. Schelter

**Affiliations:** a Roy and Diana T. Vagelos Laboratories , Department of Chemistry , University of Pennsylvania , 231 South 34th St. , Philadelphia , Pennsylvania 19104 , USA . Email: schelter@sas.upenn.edu ; Tel: +1 215-898-8633

## Abstract

A series of alkali metal cerium diphenylhydrazido complexes, M_*x*_(py)_*y*_[Ce(PhNNPh)_4_], M = Li, Na, and K, *x* = 4 (Li and Na) or 5 (K), and *y* = 4 (Li), 8 (Na), or 7 (K), were synthesized to probe how a secondary coordination sphere would modulate electronic structures at a cerium cation. The resulting electronic structures of the heterobimetallic cerium diphenylhydrazido complexes were found to be strongly dependent on the identity of the alkali metal cations. When M = Li^+^ or Na^+^, the cerium(iii) starting material was oxidized with concomitant reduction of 1,2-diphenylhydrazine to aniline. Reduction of 1,2-diphenylhydrazine was not observed when M = K^+^, and the complex remained in the cerium(iii) oxidation state. Oxidation of the cerium(iii) diphenylhydrazido complex to the Ce(iv) diphenylhydrazido one was achieved through a simple cation exchange reaction of the alkali metals. UV-Vis spectroscopy, FTIR spectroscopy, electrochemistry, magnetic susceptibility, and DFT studies were used to probe the oxidation state and the electronic changes that occurred at the metal centre.

## Abstract 2

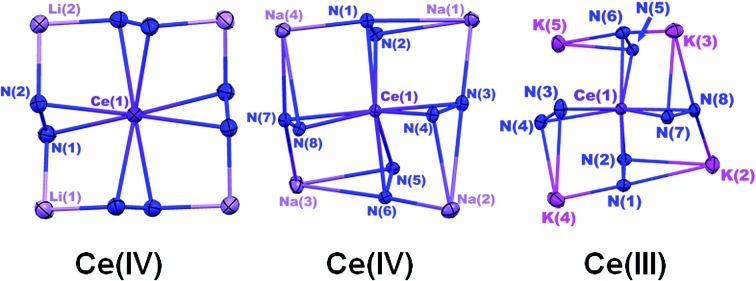
A series of alkali metal cerium diphenylhydrazido complexes, M_*x*_(py)_*y*_[Ce(PhNNPh)_4_], M = Li, Na, and K, *x* = 4 (Li and Na) or 5 (K), and *y* = 4 (Li), 8 (Na), or 7 (K), were synthesized to probe how a secondary coordination sphere would modulate electronic structures at a cerium cation.

## Introduction

Cerium is unique among the lanthanides because of its accessible +4 oxidation state (*E*°(Ce^IV/III^) = 1.40 V *vs.* Fc/Fc^+^).^1^–^3^ Considering its standard reduction potential, Ce(iv) complexes are best known as one-electron oxidants in inorganic and organic syntheses, as well as in materials chemistry.^4^–^16^ For example, ceric ammonium nitrate (CAN), has been used in water oxidation, oxidation of alcohols, oxidative carbon–carbon coupling reactions, and in oxidative deprotection of ketones and acetals.^4^,^10^ In materials chemistry cerium has been used in both oxidative and reductive contexts. Cerium(iv) dioxide (ceria) and related materials are applied in catalytic redox cycling devices, such as in fuel cells,^15^,^17^–^21^ active supports in 3-way catalytic converters,^22^–^24^ for the water gas shift reaction,^25^–^28^ and in heterogeneous catalysis for organic reactions and fuel production.^11^,^12^,^29^–^32^

We have studied the electrochemical behaviour of a variety of cerium complexes,^33^,^34^ and found that, despite the isolated nature of the cerium 4*f*^1^ electron, electron donating ligands shift the Ce(IV/III) redox potential to reducing values, *e.g. E*_1/2_(Ce^IV/III^[2-(^*t*^BuNO)py]_4_) = –1.95 V *vs.* Fc/Fc^+^, where 2-(^*t*^BuNO)py is *N-tert*-butyl-*N*-2-pyridylnitroxide.^33^,^35^ To further expand cerium redox chemistry we recently have focused on understanding the thermodynamic and kinetic factors that underlie cerium redox reactions. We demonstrated that in the cerium heterobimetallic frameworks, [M_3_(THF)_*n*_][Ce(BINOLate)_3_] M = Li, Na, K, Cs and BINOL = (*S*)-1,1′-bi-2-naphthol, the secondary coordination sphere, namely the identity of M^+^, impacted the rates and product outcomes of electron transfer (ET) reactions.^36^,^37^ Given these observations, we were compelled to investigate cerium heterobimetallic complexes with redox active ligands to express and modulate cerium–ligand intramolecular redox chemistry.

Herein, we report that the choice of alkali metal cation in the complexes M_*x*_(py)_*y*_[Ce(PhNNPh)_4_], M = Li, Na, and K, *x* = 4 (Li and Na) or 5 (K), and *y* = 4 (Li), 8 (Na), or 7 (K), resulted in variable electronic structures. Our results showed the smaller, harder alkali metal cations Li^+^ and Na^+^ stabilized the tetravalent cerium cation whereas the softer K^+^ formed a cerium(iii) complex. To the best of our knowledge, these results are the first examples of the use of secondary coordination sphere effects to modulate the oxidation state of a lanthanide cation.

## Results and discussion

### Synthesis and structural characterization of **1–3**

**Scheme 1:**
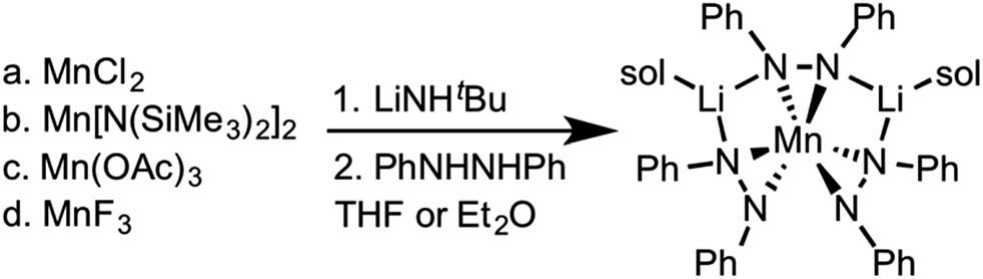
Synthesis of the lithium manganese diphenylhydrazido complex.^40^

Zdilla and coworkers recently reported a Li^+^ heterobimetallic diphenylhydrazido complex that effectively stabilized high valent Mn(iv) cations despite the reducing character of the ligand and the oxidizing character of Mn(iv) (see Scheme 1).^38^–^40^ Intrigued by these results and the relative scarcity of electrochemical properties reported for anionic nitrogen donors at cerium,^41^ we hypothesized that 1,2-diphenyl hydrazido ligands would similarly form cerium complexes with secondary structures governed by alkali metal cations.

**Scheme 2:**
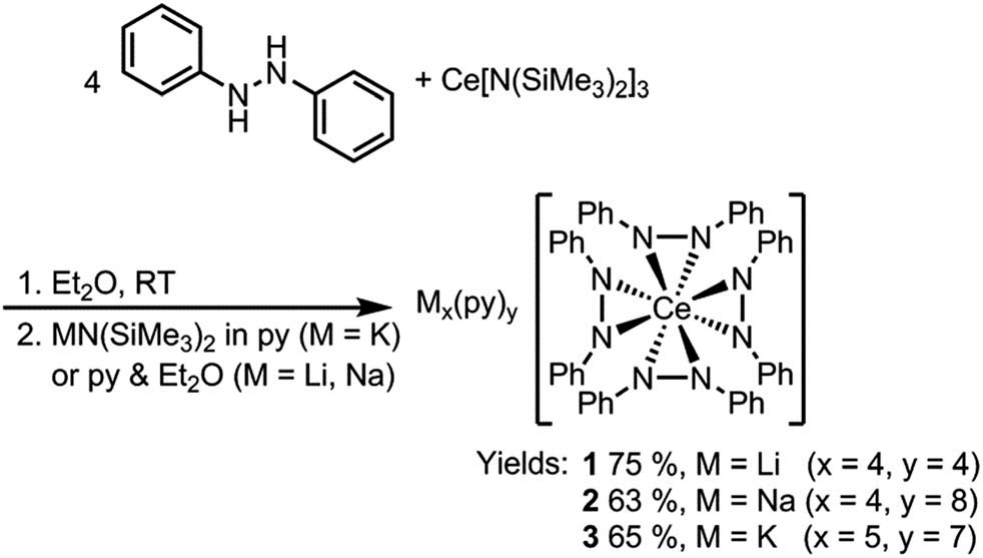
Syntheses of complexes **1**, **2**, and **3**.

Dark purple Li_4_(py)_4_[Ce(PhNNPh)_4_] (**1**) and Na_4_(py)_8_[Ce(PhNNPh)_4_] (**2**) were synthesized by layered reactions of Ce[N(SiMe_3_)_2_]_3_ with 4 equiv. 1,2-diphenylhydrazine and 4 equiv. MN(SiMe_3_)_2_, M = Li or Na, in a mixture of Et_2_O and pyridine. The yields for **1** and **2** were 75% and 63% respectively (Scheme 2). Because of the poor solubility of K_5_(py)_7_[Ce(PhNNPh)_4_] (**3**) in Et_2_O–pyridine mixtures, **3** was prepared in neat pyridine by reaction of Ce[N(SiMe_3_)_2_]_3_ with 4 equiv. 1,2-diphenylhydrazine and 5 equiv. KN(SiMe_3_)_2_. Complex **3** was isolated as dark brown needles in 65% yield following crystallization from a concentrated pyridine solution of the reaction mixture layered with hexanes.

**Fig. 1:**
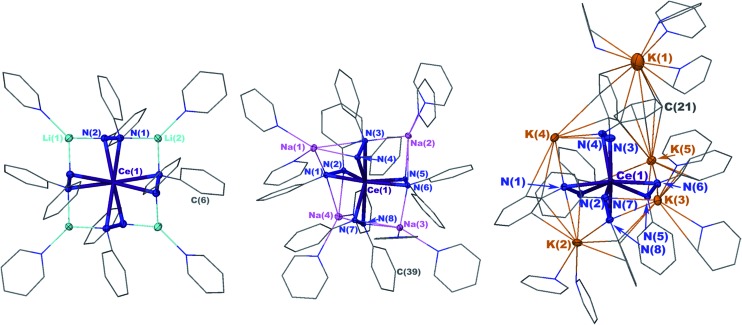
30% probability thermal ellipsoid plots of Li_4_(py)_4_[Ce(PhNNPh)_4_] (**1**) (left), Na_4_(py)_8_[Ce(PhNNPh)_4_] (**2**) (middle), and K_5_(py)_7_[Ce(PhNNPh)_4_] (**3**) (right) with the phenyl and pyridine rings shown in wire frame. Hydrogen atoms were omitted for clarity. Selected bond distances for **1** (Å): Ce(1)–N(1) 2.4408(13), Ce(1)–N(2) 2.4199(13), N(1)–N(2) 1.451(2), Li(2)–N(1) 2.018(3), Li(2)–N(1′) 2.018(3), Li(1)–N(2) 1.995(3). Selected bond distances for **2** (Å): Ce(1)–N(1) 2.390(3), Ce(1)–N(2) 2.373(2), N(1)–N(2) 1.462(3), Na(1)–N(1) 2.853(3), Na(1)–N(2) 2.630(3), Na(1)–N(3) 2.535(3). Selected bond distances for **3** (Å): Ce(1)–N(1) 2.564(3), Ce(1)–N(2) 2.480(4), N(1)–N(2) 1.465(5), K(2)–N(1) 3.044(4), K(2)–N(2) 2.877(4).

X-ray crystal structures revealed that **1** and **2** both formed formally Ce(iv) complexes by charge balance, with four dianionic diphenylhydrazido ligands and four alkali metal cations per cerium cation in the formula unit (Fig. 1). Within the structures, the alkali metal cations bridged neighbouring 1,2-diphenylhydrazido units. Surprisingly, **3** formed an extended coordination polymer in which the potassium ions interacted both intramolecularly through bridging neighbouring hydrazido ligands and intermolecularly through K–arene interactions within the ligands at K(4) and K(5) (Fig. 1). The most notable difference in the structure of **3**, however, was the presence of an additional K^+^ cation per formula unit, indicating that **3** was a formally Ce(iii) complex.

The N–N bond lengths in complexes **1**, **2**, and **3** were consistent with single bonds ranging from 1.451(2)–1.466(3) Å (Table 1).^39^,^42^–^46^ The Ce–N distances for **1** ranged from 2.4199(13)–2.4408(14) Å while those for **2** were slightly shorter at 2.373(2)–2.398(2) (Table 1). The shortened Ce–N distances for **2** compared to **1** were consistent with the stronger Lewis acidity of Li^+^ cations in **1***versus* Na^+^ cations in **2**. The Li^+^ cations reduced the relative charge density at the nitrogen atoms for binding with the cerium cation, compared to the Na^+^ cation in **2**. This effect was reversed in **3**, however, with Ce–N bonds ranging from 2.449(3)–2.636(4), in support of bonding to the larger cerium(iii) cation in that complex. This set of bond distances also indicated that a change in secondary coordination sphere caused a change in cerium electronic structure.

**Table 1:** Unique Ce(1)–N and N–N bonds and the tabulation of *τ*_4_ parameters for complexes **1**, **2**, and **3** measured by X-ray crystallography or DFT calculations

Complex	Ce(1)–N(*x*) (exp, Å)	Ce(1)–N(*x*) (calc, Å)^*a*^	N–N (exp, Å)	N–N (calc, Å)^*a*^	*τ*_4_ (exp)^*b*^	*τ*_4_ (calc)^*a*^ ^,^^*b*^
**1**	2.4408(14)	2.464	1.451(2)	1.441	0.110	0.000
	2.4199(13)					
**2**	2.390(3)	2.439	1.462(3)	1.441	0.663	0.498
	2.373(2)	2.443	1.461(3)			
	2.380(3)		1.457(3)			
	2.381(2)		1.466(3)			
	2.398(2)					
	2.374(2)					
	2.397(2)					
	2.394(2)					
**3**	2.564(3)	2.582	1.465(5)	1.448	0.773	0.709
	2.480(4)	2.488	1.449(5)			
	2.415(4)		1.459(5)			
	2.636(4)		1.456(5)			
	2.499(3)					
	2.482(4)					
	2.449(3)					
	2.494(3)					

^*a*^Pyridine was replaced with OMe_2_ in the calculated structures, resulting in the following calculated complexes: Li_4_(OMe_2_)_4_[Ce(PhNNPh)_4_], Na_4_(OMe_2_)_4_[Ce(PhNNPh)_4_], and K_4_(OMe_2_)_4_[Ce(PhNNPh)_4_]^–^.

^*b*^ *τ*_4_ values were calculated using the angles formed from the centroids of the N–N bonds and the cerium cations.

To better understand the structural differences between complexes **1–3**, we established the geometrical changes to the Ce primary coordination sphere using shape parameters for eight coordinate complexes (Table S1, see the ESI†).^47^–^49^ The shape parameters showed that each structure could be described by distinct eight-coordinate geometries with complexes **1** and **2** resembling dodecahedra (*D*_2d_), and complex **3** resembling a square antiprism (*D*_4d_). However, complexes **1–3** were heavily distorted from the idealized structures described by shape parameters, which made it difficult to express the three-dimensional structural differences between each of the complexes. Because of the distortions, the parameter *τ*_4_, which typically indicated the degree of planarity in four-coordinate structures, was found to be a more convenient metric to describe the system (Fig. 1 and Table 1).^50^ The four centroids between the N–N bonds of the ligands were used to calculate *τ*_4_ parameters for each complex, where a *τ*_4_ value of 0 indicated a planar distribution of the centroids and a value of 1 indicated a tetrahedral distribution of the centroids.^50^ The *τ*_4_ values revealed that as the ionic radius of the alkali metal cation increased, the geometry of the structure changed from pseudo-planar (**1**) *τ*_4_ = 0.110 to pseudo-tetrahedral (**3**) *τ*_4_ = 0.773 (Table 1 and Fig. 1).

**Scheme 3:**
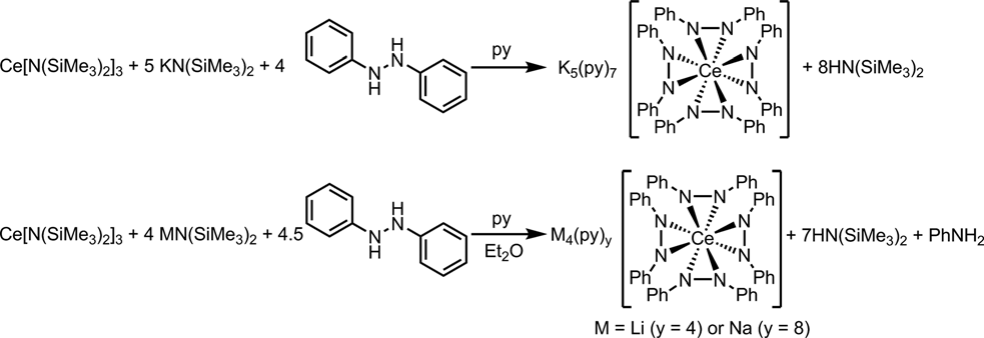
The balanced chemical equation for the formation of **3** (top), and **1** and **2** (bottom).

We also examined the reaction mixtures of **1** and **2** by NMR spectroscopy for the reduction product from the spontaneous oxidation of Ce(iii) to Ce(iv) (Scheme 3). Reductive cleavage of 1,2-diphenylhydrazine (*E*_1/2_ = –1.7 V *versus* Fc/Fc^+^ in DMSO)^51^ by metal complexes, including f-block metals, has been established to yield aniline, metal anilides, and metal diphenylamides.^38^–^40^,^52^–^62^ We postulated the balanced equation in Scheme 3 was at work in the synthesis of complexes **1** and **2**, where the electron transfer occurred from a Ce(iii) cation to 0.5 equiv. 1,2-diphenylhydrazine to form aniline.^40^ Indeed, aniline was detected by ^1^H NMR spectroscopy of the reaction mixture of complex **1** (see the ESI, Fig. S1–S4†). As expected, the reaction mixture for complex **3** did not show any evidence of aniline formation.

The reduction of 1,2-diphenylhydrazine by Ce(iii) was surprising as the reduction potential of 1,2-diphenylhydrazine was thermodynamically inaccessible.^3^,^51^ However, coordination of substrates to one or more Lewis acids had been shown to promote electron transfer.^63^–^74^ Fukuzumi and coworkers quantified the effect of Lewis acid coordination to O_2_ and its reduction with (TPP)Co (TPP = tetra-*p*-tolylporphyrin).^73^ Similarly, the reduction of 1,2-diphenylhydrazine by Ce(iii) in the formation of complexes **1–3** was determined to be dependent on the Lewis acidity of the countercation, where K^+^ was not sufficiently Lewis acidic to promote the reduction of 1,2-diphenylhydrazine.

### Magnetic, spectroscopic, and electrochemical characterization

**Fig. 2:**
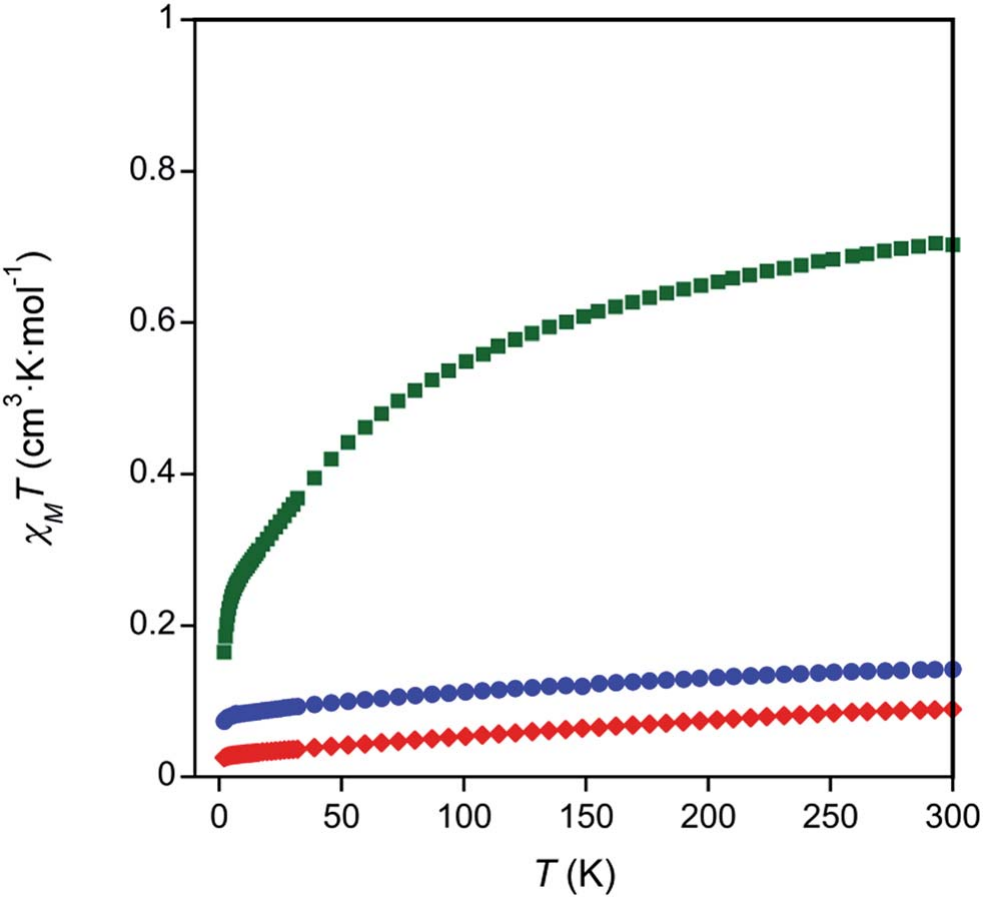
Temperature dependent magnetic data for complexes **1** (blue circles), **2** (red diamonds), and **3** (green squares).

To investigate the valences of complexes **1–3**, magnetism and Ce L_III_-edge XAS spectroscopy studies were performed. The oxidation state of complex **3** was corroborated with room temperature magnetic susceptibility measurements using Evans' method^75^,^76^ and solid state SQUID magnetometry (Table S3 and Fig. S5–S7 in the ESI,† Fig. 2). The room temperature *χT* products measured by both techniques, *χT* = 0.57 cm^3^ K mol^–1^ by Evans' method and *χT* = 0.70 cm^3^ K mol^–1^ by SQUID magnetometry, were similar to other reported Ce(iii) complexes.^77^ Temperature dependent susceptibility plots of Ce(iii) complexes typically show a decrease in the *χT* product at low temperatures due to thermal depopulation of crystal field levels,^78^–^80^ which was also observed in our data (Fig. 2). The low temperature *χT* product was also similar to previously reported Ce(iii) complexes.^77^ Complexes **1** and **2** showed only a small paramagnetic shift in solution when Evans' method was applied (0.01 ppm compared to 0.1 ppm seen in complex **3**, see ESI, Table S3†). Similarly, the room temperature *χT* products obtained by SQUID magnetometry were small, *χT* = 0.18 cm^3^ K mol^–1^ and *χT* = 0.089 cm^3^ K mol^–1^ for complexes **1** and **2** respectively (Fig. 2). The paramagnetic responses from complexes **1** and **2** were attributed to a small amount of paramagnetic impurity observed consistently over multiple measurements. Consistent with the magnetic data, the UV-Vis absorption spectra of **1** and **2** showed broad ligand-to-metal charge transfer bands, which are defining features in many Ce(iv) complexes (Fig. S26–S27, ESI†).^36^,^81^ Ce L_III_-edge spectroscopic data were determined to be unreliable due to the instability of the complexes in the sample matrix (Fig. S8†).

IR spectroscopy was performed to probe the change in N–N stretching frequency that was expected to accompany a change in formal oxidation state. We expected that the N–N stretching modes would increase in energy from complex **1** to **3** based on the Lewis acidity of both the Ce ion and the alkali metal cation in each complex. Compared to Ce(iii) cations, the more Lewis acidic Ce(iv) cation was expected to accept more electron density through σ-bonding with the N–N units, reducing the electron density in the N–N bonds (see Fig. S38 in the ESI†). We also expected the alkali metal cations would similarly withdraw more electron density with increasing Lewis acidity. DFT calculations were used to identify the vibrational modes in the experimental spectra (see DFT section for further details). DFT computed vibrational spectra typically overestimate the energies of the vibrational modes,^82^–^86^ but apart from this overestimation, the calculated and experimental spectra were in good agreement (Table 2, see Fig. S23–S25 in the ESI†).

**Table 2:** Energies of the N–N stretch vibrational modes of **1**, **2**, and **3** determined experimentally and by calculations. Spectrometer errors for the experimental spectra = ±0.034 cm^–1^.^87^ The calculated values were not scaled, see text for details.^82^–^86^

Complex	N–N stretch (cm^–1^, sol = py)	N–N stretch (cm^–1^, sol = OMe_2_)
Li_4_(sol)[Ce(PhNNPh)_4_] (**1**)	1255	1284
Na_4_(sol)[Ce(PhNNPh)_4_] (**2**)	1257	1289
K_4_(OMe_2_)_4_[Ce(PhNNPh)_4_]^–^ (**3-**OMe_2_)	—	1303
{K_5_(py)_7_[Ce(PhNNPh)_4_]}_*n*_ (**3**)	1265	—

The intense absorption band of the N–N stretch in complexes **1**, **2**, and **3** in both the experimental and calculated spectra allowed for assignment of that vibrational mode (see ESI, Fig. S23–S25†). The Ce–N vibrational modes were difficult to unambiguously assign because of their low intensities and overlap with ligand features. Complex **3** showed a ∼10 cm^–1^ increase in energy of the N–N stretching mode from complexes **1** and **2**, consistent with a change in cerium oxidation state. Because the N–N stretching mode found in complexes **1** and **2** differed by only 2 cm^–1^ despite the change in Lewis acidity between Li^+^ and Na^+^, the more significant factor to the increase in energy between these complexes was evidently the oxidation state at the Ce cation. Overall, based on the spectroscopic and magnetic measurements, the oxidation state of the cerium metal centre was evidently impacted by the identity of the alkali metal cation in the secondary coordination sphere.

To better understand how the alkali metal cation in the secondary coordination sphere influenced the redox properties of the cerium metal centre, cyclic voltammetry was performed on complexes **1–3** (see Fig. S32–S37 in the ESI†). Because of the instability of complexes **1** and **2** in coordinating solvents and the insolubility of complex **3** in non-coordinating solvents, the electrochemistry of complexes **1** and **2** was performed in fluorobenzene, and in THF for complex **3**. Beginning the scans from the rest potentials in each case, the Ce^IV/III^ reduction waves (in the cases of **1** and **2**) and oxidation wave (in the case of **3**) were reversible for complexes **1–3**, where the difference in the anodic and cathodic waves were Δ*E* = 50 mV for complex **3** in THF, and Δ*E* = 60–70 mV for complexes **1** and **2** in fluorobenzene. The electrochemical reversibility of the Ce^IV/III^ redox event indicated that outer sphere electron transfer between cerium and the electrode was rapid in all three complexes, and that there was little ligand reorganization upon oxidation or reduction of the cerium cation in any geometry.

In order to compare the electrochemical potentials of complexes **1–3**, we used 1,2-diphenylhydrazine, which was measured in both solvents, to normalize the influence of the solvent on the electron transfer between the electrode and the analyte. The *E*_1/2_ values for the Ce^IV/III^ reduction of complexes **1** and **2** were centred at –1.93 V and –1.88 V *versus* Fc/Fc^+^ respectively. Following the normalization for the difference in solvents, the *E*_1/2_ of the Ce^III/IV^ oxidation of complex **3** was –2.02 V *versus* Fc/Fc^+^. The negative reduction potentials indicated that the Ce(iv) oxidation state was strongly stabilized by the hard, anionic PhNNPh^2–^ ligand. Use of electron-rich anionic N-donors had been shown to support the Ce(iv) oxidation state previously.^33^ Thus, despite its measurement as a slightly more potent reductant than **1** and **2**, complex **3** was isolated as a Ce(iii) complex.

### Ce(iv) and cation exchange reactions

**Scheme 4:**
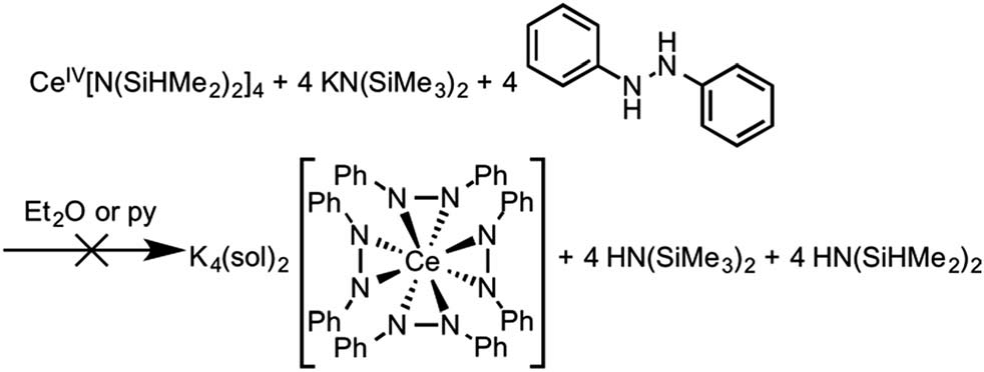
Attempted synthesis of K_4_(sol)_2_[Ce(PhNNPh)_4_] starting from a Ce(iv) precursor. Instead, K_5_(py)_7_[Ce(PhNNPh)_4_] (**3**) formed as a result of this reaction (Fig. S22, ESI†).

Considering the oxidation potential of complex **3**, we reasoned a potassium-supported Ce(iv) diphenylhydrazido analogue should be accessible. In an effort to isolate a Ce(iv) diphenylhydrazido complex with K^+^ cations in the secondary coordination sphere, we reacted 1,2-diphenylhydrazine with a formally Ce(iv) protonolysis starting material, Ce[N(SiHMe_2_)_2_]_4_.^88^,^89^ The addition of 1,2-diphenylhydrazine to the Ce(iv) starting material, however, resulted in immediate reduction (Scheme 4) and subsequent reaction with KN(SiMe_3_)_2_ formed complex **3**, which was detected by ^1^H NMR spectroscopy. Adding 1,2-diphenylhydrazine to a mixture of KH and Ce[N(SiHMe_2_)_2_]_4_ also resulted in the formation of complex **3**. Presumably, 0.5 equiv. azobenzene was the other product that formed in the course of the redox reaction, but this side product was not detected by ^1^H NMR spectroscopy (see Fig. S22, ESI†). Thus, the presence of alkali metal cations in the secondary coordination sphere were essential in stabilizing the Ce(iv) oxidation state. The alkali metals in solution structurally supported the resultant cerium diphenylhydrazido complex as well as modulated the electronics of the ligands in order to stabilize Ce(iv) and destabilize the ligand oxidation. These observations were consistent with those of Zdilla and coworkers, who disclosed that, upon removing the Li^+^ cations in the cluster Li_4_Mn_4_(μ_3_-N^*t*^Bu)_3_(N^*t*^Bu)(N), the Mn(v) cations reductively eliminated azo-*tert*-butane.^90^

**Scheme 5:**
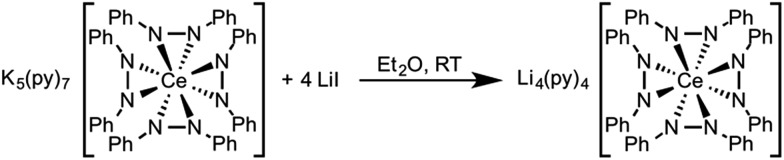
Metathesis reaction of complex **3** with 4 equiv. of LiI.

Based on the similar cerium electrochemistry of complexes **1** and **3** and the Ce–N and M–N (M = Li^+^ and K^+^) bond enthalpies, we expected that complex **3** should convert to complex **1** through a cation exchange reaction (Scheme 5). The successful metathesis of LiI with complex **3** in diethyl ether demonstrated that the oxidation state of cerium could be influenced by a change in the secondary coordination sphere (Fig. S15, ESI†). This reaction likely occurred through a Li^+^ promoted reduction of the 1,2-diphenylhydrazido ligand as well. By ^1^H NMR spectroscopy, the reaction appeared to proceed cleanly, but the percent conversion was consistently overestimated. Presumably the resonances of side-products present in the reaction overlapped with the desired products' resonances. Cation exchange reactions of **2** with KI were also successful, where complex **3** was the only product evident by ^1^H NMR spectroscopy (Fig. S20–S21 in the ESI†). In this case, the 1,2-diphenylhydrazido evidently acted as the reductant to form Ce(iii) and azobenzene. A complex mixture formed when cation exchange of complex **1** with KI was attempted (Fig. S17–S19†). The complex mixture observed in this reaction could be as a result of the smaller lattice energy of LiI formation, the poorer solubility of KI, and the weaker M–N interaction with larger alkali metal cations.^91^,^92^

## Conclusions

We have shown that the secondary coordination sphere about cerium 1,2-diphenylhydrazido complexes influenced both the primary coordination geometry and the oxidation state of the cerium metal centre, making these complexes the first example of a secondary coordination sphere influencing the oxidation state of f-element complexes. The alkali metals in the secondary coordination sphere both facilitated the reduction of the 1,2-diphenylhydrazine to produce a high-valent Ce(iv) complex, and structurally stabilized the high-valent Ce(iv) complexes. Furthermore, a cation exchange reaction with simple alkali metal salts had changed the oxidation state at the cerium ion. The use of multiple Lewis acids could be a general strategy to access high valent metal oxidation states. Electronic and steric changes to the primary coordination sphere in this system are currently under investigation.

## Experimental section

### General methods

Unless otherwise indicated all reactions and manipulations were performed under an inert atmosphere (N_2_) using standard Schlenk techniques or in a Vacuum Atmospheres, Inc. Nexus II drybox equipped with a molecular sieves 13X/Q5 Cu-0226S catalyst purifier system. Glassware was oven-dried overnight at 150 °C prior to use. ^1^H, ^13^C, and ^7^Li NMR spectra were obtained on a Bruker DMX-300, on a Bruker DMX-360, or on a Bruker DRX-400 Fourier transform NMR spectrometer at 300, 360, and 400 MHz respectively. Chemical shifts were recorded in units of parts per million downfield from residual proteo solvent peaks (^1^H), or characteristic solvent peaks (^13^C). The ^7^Li spectra were referenced to external solution standards of LiCl in H_2_O. Evans' method was performed on a Bruker BioDRX-500 Fourier transform NMR spectrometer at 500 MHz. Hexamethylcyclotrisiloxane was used as the internal standard for Evans's method experiments. Elemental analyses were performed at the University of California, Berkeley Microanalytical Facility using a Perkin-Elmer Series II 2400 CHNS analyzer. UV-vis-NIR absorption measurements of complexes were performed using a PerkinElmer 950 UV-vis/NIR Spectrophotometer. One mm path length screw cap quartz cells were used with a blank measured before each run. The infrared spectra were obtained from 400–4000 cm^–1^ using a PerkinElmer 1600 series infrared spectrometer. FTIR solution spectra were first collected with a background of air, and then the solvent spectrum was subtracted using PerkinElmer software. GC/MS Spectrometry was performed using an Agilent 5937 GC/MS spectrometer with the CI method of ionization. Diethyl ether was used as the volatile solvent and He as the carrier gas.

### Materials

Tetrahydrofuran, diethyl ether, hexane, fluorobenzene, and pentane were purchased from Fisher Scientific. The solvents were sparged for 20 min with dry N_2_ and dried using a commercial two-column solvent purification system comprising columns packed with Q5 reactant and neutral alumina respectively (for hexane and pentane), or two columns of neutral alumina (for THF, diethyl ether, and fluorobenzene). Pyridine, also purchased from Fisher Scientific, was freeze–pump–thawed for 4 cycles and stored over 4 Å molecular sieves for three days before use. Deuterated solvents were purchased from Cambridge Isotope Laboratories, Inc. Pyridine-*d*_*5*_ was stored over 4 Å molecular sieves for three days before use, and benzene-*d*_*6*_ was dried and stored over potassium for 2 days before use. Ce[N(SiMe_3_)_2_]_3_ 93 and Ce[N(SiHMe_2_)_2_]_4_ 88,89 were prepared following published procedures. Li[N(SiMe_3_)_2_] (Acros) was recrystallized from hot pentane prior to use. K[N(SiMe_3_)_2_] (Acros) and Na[N(SiMe_3_)_2_] (Acros) were used as purchased. Hydrazobenzene (Sigma Aldrich, MP Biochemicals, Alfa Aesar) was sublimed under reduced pressure first at 60 °C to remove azobenzene, and then at 105 °C for further purification prior to use. Alternatively, the hydrazobenzene was purified by fractional recrystallization of hydrazobenzene dissolved in toluene and layered with pentane.

### Electrochemistry

Voltammetry experiments (CV and DPV) were performed using a CH Instruments 620D Electrochemical Analyzer/Workstation and the data were processed using CHI software v 9.24. All experiments were performed in an N_2_ atmosphere drybox using electrochemical cells that consisted of a 4 mL vial, glassy carbon (3 mm diameter) working electrode, a platinum wire counter electrode, and a silver wire plated with AgCl as a quasi-reference electrode. The working electrode surfaces were polished prior to each set of experiments, and were periodically replaced to prevent the build-up of oxidized product on the electrode surfaces. Potentials were reported *versus* ferrocene (Fc), which was converted from cobaltocene for calibration at the end of each run.^3^ Solutions employed during CV studies were ∼3 mM in analyte. For electrochemistry collected in fluorobenzene, the solution was 100 mM in [^*n*^Bu_4_N][B(3,5-(CF_3_)_2_-C_6_H_3_)_4_] ([^*n*^Bu_4_N][BAr^F^_4_]), and electrochemistry collected in THF had 100 mM in [^*n*^Pr_4_N][B(3,5-(CF_3_)_2_-C_6_H_3_)_4_] ([^*n*^Pr_4_N][BAr^F^_4_]) as the electrolyte. All data were collected in a positive-feedback IR compensation mode. Scan rate dependences of 25–1000 mV s^–1^ were performed to determine electrochemical reversibility.

### Magnetism

Magnetic data were collected on a Quantum Design Multi-Property Measurement System (MPMS-7) with a Reciprocating Sample Option at 1 T from 2 to 300 K and at 2 K and 300 K from 0 to 7 T. Quartz wool was dried at 250 °C prior to use, plastic drinking straws were evacuated overnight prior to use. The plastic drinking straws themselves were used as the sample holders through heat sealing the plastic tubing. The plastic drinking straws were heat sealed at one end in a glovebox. Then a ground sample was loaded into the straw and capped with ∼10 mg of quartz wool. The other end of the plastic drinking straw was then sealed through application of heat, forming a pouch that contained the sample and the quartz wool. The sample and wool were weighed to the nearest 0.1 mg on a calibrated and levelled Mettler-Toledo AL-204 analytical balance. Corrections for the intrinsic diamagnetism of the samples were made using Pascal's constants.^94^ Data were collected on two independently prepared samples to ensure reproducibility.

### X-ray absorption spectroscopy

Ce L_III_-edge XANES data were collected at the Stanford Synchrotron Radiation Lightsource, beamline 11-2, using a Si 220 (phi = 0) double monochromator that was detuned to 20% in order to reduce harmonic contamination. The resulting data have an energy resolution of 3.2 eV. Data were collected in transmission, using a CeO_2_ reference to calibrate the energy scale, setting the first inflection point of the CeO_2_ absorption to 5723 eV. A linear pre-edge background was subtracted and the data were subsequently normalized at 5800 eV.

Since the compounds are extremely sensitive to oxygen, each sample was ground into a powder, mixed with dry boron nitride as a diluent, and then packed into the slots of a machined aluminum sample holder. Aluminized mylar was affixed to the holder with an indium-wire seal. After packaging, the samples were transported in dry nitrogen-filled containers to the beamline. Sample holders were quickly transferred to the vacuum chamber, exposing the sealed holders to air for less than thirty seconds before pumping out the chamber and collecting the data under vacuum.

### X-ray crystallography

X-ray intensity data were collected on a Bruker APEXII CCD area detector employing graphite-monochromated Mo-Kα radiation (*λ* = 0.71073 Å) at a temperature of 143(1) K. In all cases, rotation frames were integrated using SAINT,^95^ producing a listing of unaveraged *F*^2^ and *σ*(*F*^2^) values which were then passed to the SHELXTL^96^ program package for further processing and structure solution on a Dell Pentium 4 computer. The intensity data were corrected for Lorentz and polarization effects and for absorption using TWINABS^97^ or SADABS.^98^ The structures were solved by direct methods (SHELXS-97).^99^ Refinement was by full-matrix least squares based on *F*^2^ using SHELXL-97.^99^ All reflections were used during refinements. The weighting scheme used was *w* = 1/[*σ*^2^(*F*_o_^2^) + (0.0907*P*)^2^ + 0.3133*P*] where *P* = (*F*_o_^2^ + 2*F*_c_^2^)/3. Non-hydrogen atoms were refined anisotropically and hydrogen atoms were refined using a riding model. Complex **1** (Li_4_(py)_4_[Ce(PhNNPh)_4_] C_68_H_60_N_12_Li_4_Ce), crystallizes in the orthorhombic space group *Fddd* with *a* = 11.4483(7) Å, *b* = 43.060(2) Å, *c* = 24.5220(14) Å, *V* = 12088.5(12) Å^3^, *Z* = 8, and *d*_calc_ = 1.333 g cm^–3^. A total of 74593 reflections were measured yielding 3485 unique reflections (*R*_int_ = 0.0381). Complex **2** (Na_4_(py)_8_[Ce(PhNNPh)_4_] C_88_H_80_N_16_Na_4_Ce) crystallizes in the triclinic space group *P*1 with *a* = 13.9504(7) Å, *b* = 16.2467(9) Å, *c* = 19.0053(11) Å, *α* = 83.136(3)°, *β* = 83.696(3)°, *γ* = 84.993(3)°, *V* = 4238.8(4) Å^3^, *Z* = 2, and *d*_calc_ = 1.249 g cm^–3^. A total of 147398 reflections were measured, 19328 of them unique reflections (*R*_int_ = 0.0680). Complex **3** (K_5_(py)_7_[Ce(PhNNPh)_4_]) C_83_H_75_N_15_K_5_Ce, crystallizes in the triclinic space group *P*1 with *a* = 15.2003(12) Å, *b* = 15.4668(12) Å, *c* = 18.0353(15) Å, *α* = 93.849(5)°, *β* = 101.333(5)°, *γ* = 107.292(5)°, *V* = 3933.7(5) Å^3^, *Z* = 2, and *d*_calc_ = 1.366 g cm^–3^. A total of 95153 reflections were measured yielding 17890 unique reflections (*R*_int_ = 0.0544).

### Computational details

All calculations were performed with Gaussian 09 Revision D.01 100 with the B3LYP hybrid DFT method. A 28-electron small core effective core potential was applied to cerium with published segmented natural orbital basis set incorporating quasi-relativistic effects,^101^–^103^ while the 6-31 G* basis set was applied to all other atoms. Geometry optimizations of **1** and **2** were based on their crystal structures, while the geometry optimization of the Ce(iv) structure of **3** was based off of the crystal structure coordinates of complex **2**. The coordinates that were input for the geometry optimizations of the anionic Ce(iii) analogues were based on the optimized geometry found for the Ce(iv) calculations **1**, **2**, and **3** with elongated Ce–N bonds. Geometry optimization was attempted with pyridine solvating the alkali metals. However, convergence could not be reached with this system. No other restrictions were placed on the systems besides the spin. All frequency calculations found no negative frequencies, except for the calculation of Li_4_(OMe_2_)_4_[Ce(PhNNPh)_4_] which had 4 small negative frequencies less than –17 cm^–1^, indicating that the optimized structures found were at an energy minimum. Molecular orbitals were rendered using Chemcraft v. 1.6.^104^

### Synthetic details and characterization

#### Synthesis of Li_4_(py)_4_[Ce(PhNNPh)_4_] (**1**)

Hydrazobenzene (0.083 g, 0.45 mmol) was dissolved in 3 mL of diethyl ether in a 20 mL scintillation vial. Ce[N(SiMe_3_)_2_]_3_•toluene (0.082 g, 0.12 mmol) was added to the mixture, resulting in a brown-orange suspension. After stirring for 1 h, the diethyl ether was removed under reduced pressure. Diethyl ether (or toluene) (5 mL) with pyridine (43 μL, 4.5 equiv.) was added to the green-yellow solid, resulting in an orange suspension. In a separate 20 mL scintillation vial, LiN(SiMe_3_)_2_ (0.076 g, 0.45 mmol) was weighed and dissolved in hexanes to form a colourless solution. The colourless hexanes solution was layered over the orange suspension. Product formation was concentration-dependent, but not stoichiometry dependent. The product could also be formed using bulk reactions but crystalline yield was lower. After 1 day, dark purple crystals formed. The crystals were collected by filtration over a medium frit, washed with hexane, and dried under reduced pressure. Yield: 0.103 g, 0.085 mmol, 74%. ^1^H NMR (360 MHz, C_6_D_6_) *δ* 7.93 (s, 2H), 7.26 (s, 4H), 6.69 (t, *J* = 7.2 Hz, 1H), 6.49 (t, *J* = 7.2 Hz, 2H), 6.36 (s, 2H), 6.23 (s, 4H), 3.13 (q, *J* = 7.0 Hz, 2H), 0.88 (t, *J* = 7.0 Hz, 3H). ^7^Li NMR (400 MHz, C_6_D_6_) *δ* 1.93 (0.2 Li), 1.47 (1 Li). ^13^C NMR (360 MHz, C_6_D_6_) *δ* 160.86, 149.31, 136.76, 129.36, 124.14, 118.49, 117.07. FTIR (C_6_D_6_) 3055.63, 2976.81, 2828.47, 2867.82, 1584.84 (vs), 1556.91, 1471.46 (vs), 1442.30 (s), 1416.36, 1296.71, 1286.64, 1254.62 (vs), 1164.28, 1118.78, 1071.37, 1026.25, 1019.52, 987.41, 869.14, 775.61, 751.14 (s), 702.66 (s), 693.13 (s), 618.17. Elemental analysis calculated for Li_4_(py)_3_(Et_2_O)[Ce(PhNNPh)_4_] C_67_H_65_CeLi_4_N_11_O: C, 66.61; H, 5.42; N, 12.75. Found: C, 66.36; H, 5.39; N, 12.61%.

#### Synthesis of Na_4_(py)_8_[Ce(PhNNPh)_4_] (**2**)

Hydrazobenzene (0.095 g, 0.52 mmol) was dissolved in 4 mL of diethyl ether in a 20 mL scintillation vial. Ce[N(SiMe_3_)_2_]_3_ (0.082 g, 0.13 mmol) was added to the mixture, resulting in a brown-orange suspension. After stirring for 1 h, the diethyl ether was removed under reduced pressure. Diethyl ether (2 mL) was added to the green-yellow solid, and then pyridine (105 μL, 10 equiv.) was added to form an orange suspension. NaN(SiMe_3_)_2_ (0.095 g, 0.52 mmol) was weighed in a separate 20 mL scintillation vial and dissolved in 1.5 mL diethyl ether. The dissolved NaN(SiMe_3_)_2_ was layered over the orange suspension. Finally, hexanes (1 mL) were layered over the two diethyl ether layers. 0.132 g (0.083 mmol) of purple needles were collected by filtration in a medium frit, washed with hexanes, and dried under reduced pressure. Product formation was concentration-sensitive but not stoichiometry sensitive. The product could also be formed using bulk reactions but crystalline yield was lower. Yield: 64%. ^1^H NMR (360 MHz, C_6_D_6_) *δ* 8.04 (s, 2H), 7.05 (s, 2H), 6.88 (t, *J* = 7.2 Hz, 1H), 6.76–6.52 (m, 2H), 6.51–6.14 (m, 2H), 5.89 (s, 1H), 3.21 (q, *J* = 7.0 Hz, 0.6H), 1.06 (t, *J* = 7.0 Hz, 1H). ^13^C NMR (360 MHz, C_6_D_6_) 161.23, 150.53, 136.39, 129.07, 124.04, 113.48. FTIR (C_6_D_6_) 3052.20, 2997.57, 2976.29, 22 868.66, 1582.74 (vs), 1547.75 (s), 1468.83 (vs), 1440.44 (s), 1296.41 (s), 1289.19 (s), 1256.80 (vs), 1162.99 (s), 1148.41, 1118.74, 1069.80, 1033.85, 1018.29, 987.09 (vs), 860.03 (s), 793.83, 746.86 (s), 702.93 (vs), 613.74, 599.51. Elemental analysis calculated for Na_4_(py)_5_(Et_2_O)[Ce(PhNNPh)_4_] C_77_H_75_CeNa_4_N_13_O: C, 64.65; H, 5.28; N, 12.73. Found: C, 64.58; H, 5.08; N, 12.70%.

#### Synthesis of K_5_(py)_7_[Ce(PhNNPh)_4_] (**3**)

Hydrazobenzene (0.095 g, 0.52 mmol) was dissolved in 4 mL of diethyl ether in a 20 mL scintillation vial. Ce[N(SiMe_3_)_2_]_3_ (0.081 g, 0.13 mmol) was added to the mixture, resulting in a brown-orange suspension. After stirring for 1 h, the diethyl ether was removed under reduced pressure. Pyridine was added to the green-yellow solid, resulting in a dark red solution, which over time formed a red suspension. KN(SiMe_3_)_2_ (0.124 g, 0.64 mmol) was weighed and added to the pyridine solution. A dark brown solution immediately formed. The solution was stirred for 1 day and then the pyridine was removed under reduced pressure. The resulting solid was redissolved in pyridine (1.5 mL) and then layered with hexanes (3 mL). Dark brown needles formed. The solid was collected by filtration on a medium frit, washed with hexanes, and dried under reduced pressure. While this reported synthesis reports the method that yielded the product most consistently, it is important to note that the formation of product was extremely conditions and concentration sensitive although not stoichiometry sensitive. Yield: 0.131 g, 0.081 mmol, 63%. ^1^H NMR (360 MHz, pyridine-*d*_*5*_) *δ* 7.003–6.89 (br d, 3H), 6.23 (br s, 1H), 5.84 (br, 1H). ^13^C NMR (360 MHz, pyridine-*d*_*5*_) 159.95, 150.77, 136.60, 129.21, 128.14, 124.56, 118.98, 111.08. FTIR (nujol) 3049.33, 2922.30, 2853.02, 1582.33 (vs), 1544.48 (s), 1465.91 (vs), 1439.18, 1377.00, 1298.18 (vs), 1265.63 (vs), 1163.72 (vs), 1149.11, 1071.64, 1030.39, 1018.91, 995.23, 986.20, 862.36, 818.34, 783.64, 746.92 (vs), 702.19 (vs), 608.58, 591.44, 524.12. Elemental analysis calculated for K_5_(py)_7_[Ce(PhNNPh)_4_] C_83_H_75_CeK_5_N_15_: C, 61.61; H, 4.67; N, 12.98. Found: C, 61.25; H, 4.32; N, 12.98%.

## Supplementary Material

Supplementary information

Crystal structure data
