# Supplementary material for: Control of cerium oxidation state through metal complex secondary structures
Source: Chem Sci. 2015 Aug 11;6(12):6925–34. doi: 10.1039/c5sc02607e (PMC5951102; doi:10.1039/c5sc02607e)
Supplement: Supplementary file 1 [file SC-006-C5SC02607E-s001.pdf]

Electronic Supplementary Material (ESI) for Chemical Science.
This journal is © The Royal Society of Chemistry 2015

Supporting Information for:

Control of Cerium Oxidation State through Metal Complex Secondary Structures

Jessica R. Levin, Walter L. Dorfner, Patrick J. Carroll, and Eric J. Schelter*

P. Roy and Diana T. Vagelos Laboratories, Department of Chemistry, University of Pennsylvania, 231 South 34th St. Philadelphia, Pennsylvania 19104, U.S.A.

Calculating τ	2
Table S1 Shape parameters	2
Table S2 Average Ce–N bond lengths and τ_4 values for experimental and calculated complexes	2
Figures S1–S4 Determining reaction stoichiometry by ^{1}H NMR	3–4
Table S3 Evans's Method Complexes **1–3**	5
Figures S5–S7 Magnetic data (SQUID) Complexes **1–3**	5–6
Figure S8 Cerium L_{III}-edge XAS spectroscopy of Complexes **1–3**	7
Figures S9–S10 Complex **1** ^{1}H, ^{7}Li, and ^{13}C NMR Spectra	8–9
Figures S11–S12 Complex **2** ^{1}H and ^{13}C NMR Spectra	10
Figures S13–S14 Complex **3** ^{1}H and ^{13}C NMR Spectra	11
Figure S15–S21 Metathesis reactions ^{1}H and ^{7}Li NMR spectra; GC/MS results	12–15
Figure S22 ^{1}H NMR spectrum of the reaction of 1,2-diphenylhydrazine with a Ce(IV) starting material	16
Figure S23 Complex **1** FTIR experimental and calculated spectra	17
Figure S24 Complex **2** FTIR experimental and calculated spectra	18
Figure S25 Complex **3** FTIR experimental and calculated spectra	19
Figure S26 Complex **1** UV-Vis absorption spectra in toluene and fluorobenzene	20
Figure S27 Complex **2** UV-Vis absorption spectra in toluene and fluorobenzene	20
Figure S28 Complex **1** UV-Vis absorption spectra in pyridine and fluorobenzene	20–21
Figure S29 Complex **2** UV-Vis absorption spectra in pyridine and fluorobenzene	21
Figure S30 UV-Vis absorption spectra of deprotonated 1,2-diphenylhydrazine in pyridine	21
Figure S31 Complex **3** UV-Vis absorption spectrum in pyridine	22
Figure S32 1,2-diphenyl hydrazine vs. complex **1** cyclic voltammogram measured in fluorobenzene	22–23
Figure S33 Complex **1** isolation scans and i_p vs. $v^{1/2}$ plot	23
Figure S34 1,2-diphenyl hydrazine vs. complex **2** cyclic voltammogram measured in fluorobenzene	23
Figure S35 Complex **2** isolation scans and i_p vs. $v^{1/2}$ plot	24
Figure S36 1,2-diphenyl hydrazine vs. complex **3** cyclic voltammogram measured in THF	24
Figure S37 Complex **3** isolation scans and i_p vs. $v^{1/2}$ plot	25
Figure S38 Molecular orbitals of Ce and N–N bonding	25
Tables S4–S6 Coordinates of optimized geometries for Ce(IV) analogues of **1**, **2**, and **3**	25–36
Tables S7–S9 Coordinates of optimized geometries for Ce(III) analogues of **1**, **2**, and **3**	36–47

Calculating τ_4:

The centroids between the four N–N bonds were calculated using Mercury software.[1]

$$\tau = \frac{360° - (\alpha + \beta)}{141°}$$

α and β represent the two largest angles θ between one N–N centroid, Ce, and another N–N centroid calculated using Mercury. 0 indicates a square planar structure while 1 implicates a tetrahedral structure. Similar results are obtained when τ is calculated using the largest angles from M–Ce–M, where M = Li, Na, or K.

Shape Parameters:

	Φ_1	Φ_2	δ_1	δ_2	δ_3	δ_4	θ_A	θ_B
**1**	4.0	4.0	15.0	15.0	40.9	40.9	72.1	79.8
**2**	2.0	3.8	30.0	32.4	34.1	35.2	48.8	48.7
**3**	12.6	25.3	20.2	32.7	35.3	36.8	45.7	45.8
D_{2d}[a]	0.0	0.0	29.5	29.5	29.5	29.5	35.2	73.5
D_{4d}[a]	24.5	24.5	0.0	0.0	52.4	52.4	57.3	57.3
Cube[a]	0.0	0.0	0.0	0.0	90.0	90.0	54.7	54.7

Table S1. Shape parameters for complexes **1**, **2**, and **3**. [a]Indicates the idealized shape parameters for a rigorous dodecahedron (D_{2d}), square antiprism (D_{4d}), and cube.[2]

**Complex**	**Ce(1)–N (avg., Å) (exp, sol = py)**	**Ce(1)–N (avg., Å) (calc, sol = OMe₂)**	**τ_4(exp)**	**τ_4(calc)**
Li_4(sol)[Ce(PhNNPh)$_4$] (**1**)	2.430(11)	2.464	0.110	0.000
Na_4(sol)[Ce(PhNNPh)$_4$] (**2**)	2.386(10)	2.441	0.663	0.498
$K_4(OMe_2)_4$[Ce(PhNNPh)$_4$] (**3⁺-OMe₂**)	_______	2.421	_______	0.837
$Li_4(OMe_2)_4$[Ce(PhNNPh)$_4$]$^-$ (**1⁻-OMe₂**)	_______	2.578	_______	0.116
$Na_4(OMe_2)_4$[Ce(PhNNPh)$_4$]$^-$ (**2⁻-OMe₂**)	_______	2.559	_______	0.514
$K_4(OMe_2)_4$[Ce(PhNNPh)$_4$]$^-$ (**3-OMe₂**)	_______	2.535	_______	0.709
$K_5(py)_7$[Ce(PhNNPh)$_4$] (**3**)	2.502(69)	_______	0.773	_______

Table S2. Ce–N average bond lengths and τ_4 values for both the experimental and calculated complexes.

Determining the stoichiometry of the reactions:

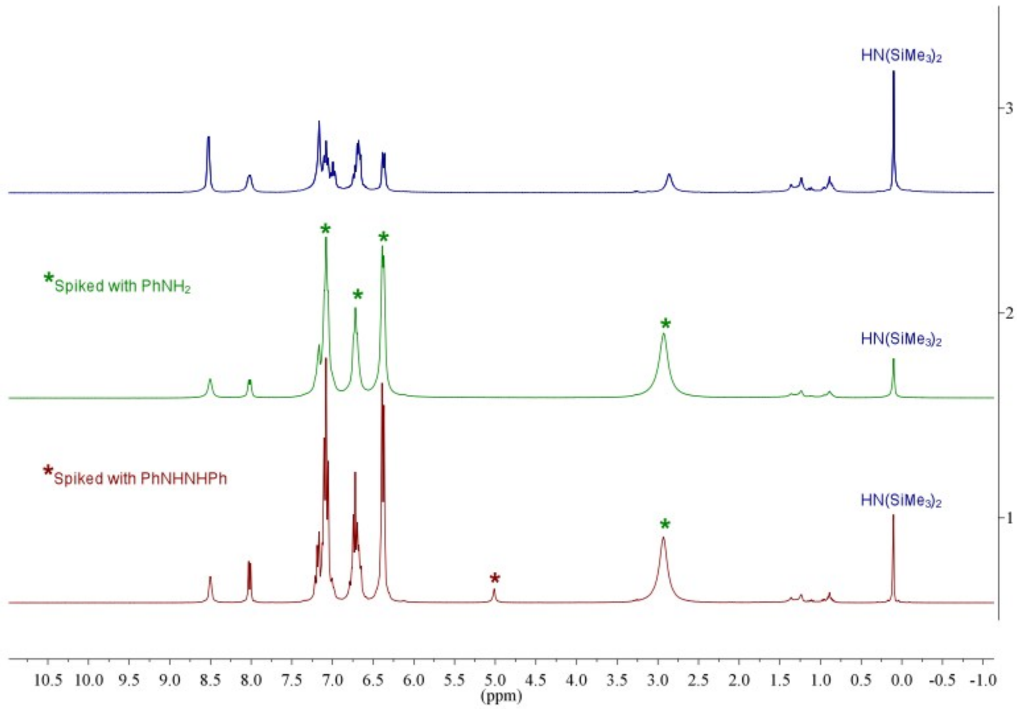

Figure S1. ^{1}H NMR spectra of (top) the filtrate from the reaction to synthesize $Li_4(py)_4[Ce(PhNNPh)_4]$ collected in C_6D_6 (middle) the filtrate spiked with aniline, and (bottom) the filtrate spiked with both aniline and 1,2-diphenylhydrazine.

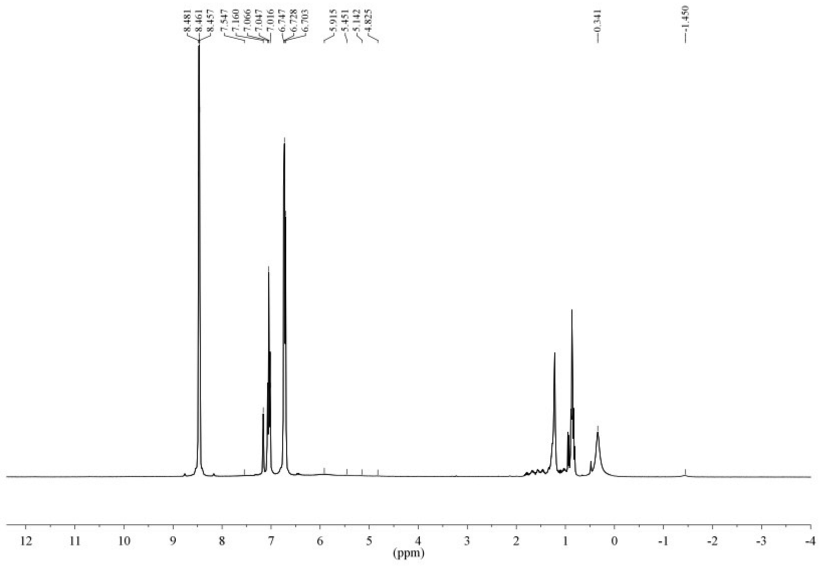

Figure S2. ^{1}H NMR spectrum of the filtrate from the reaction to synthesize $K_5(py)_7[Ce(PhNNPh)_4]$ collected in C_6D_6.

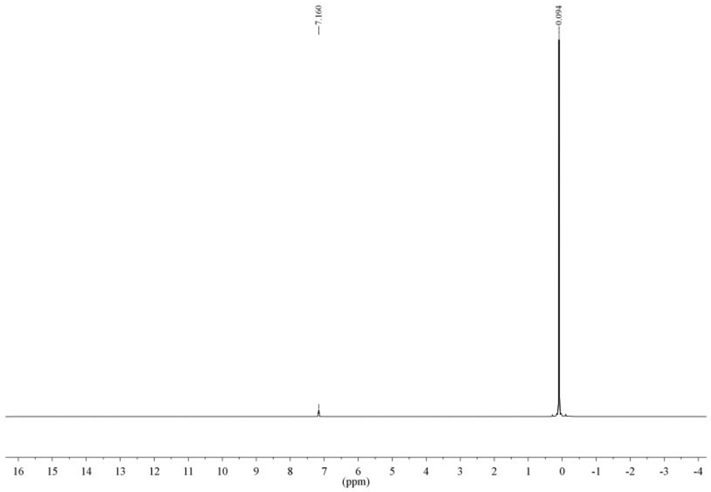

Figure S3. ^{1}H NMR spectrum in C_6D_6 of hexamethyldisilazane.

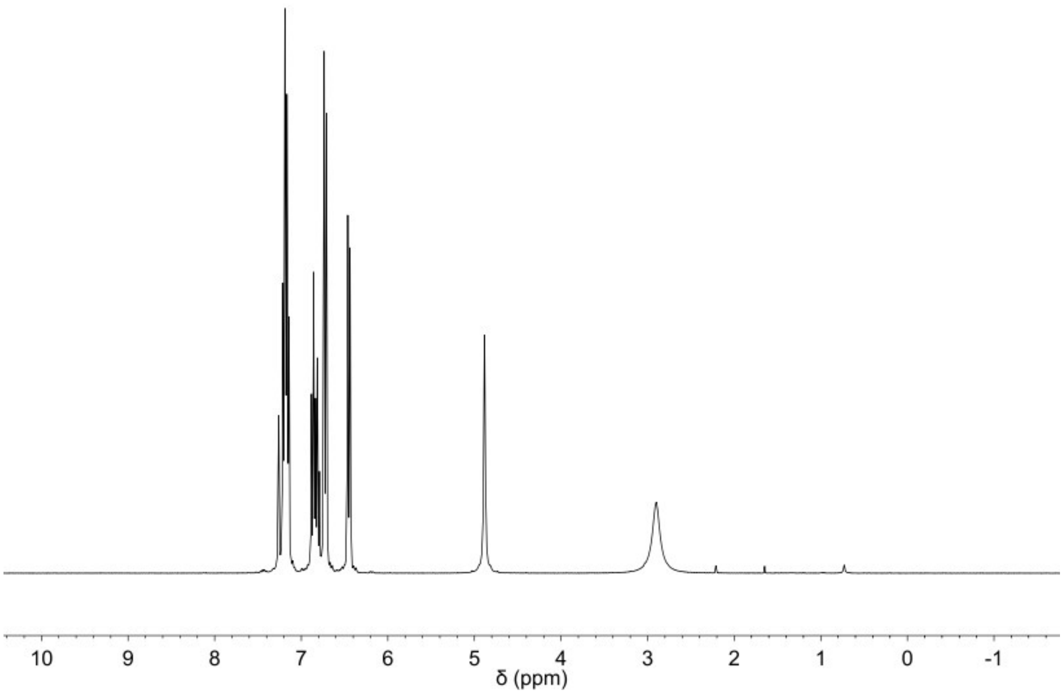

Figure S4. ^{1}H NMR spectrum in C_6D_6 of a mixture of aniline and 1,2-diphenylhydrazine.

Complex	Solvent	Concentration (M)	Δδ (ppm)	μ_{eff} (μ_B)
**1**	Toluene-d_8	0.014	0.011	1.46
**1**	Toluene-d_8	0.020	0.013	1.48
**2**	Toluene-d_8	0.012	0.010	1.54
**2**	Toluene-d_8	0.026	0.015	1.55
**3**	Pyridine-d_5	0.028	0.126	2.14
**3**	Pyridine-d_5	0.036	0.148	2.10

Table S3. Table of Evans' method results for complexes **1**, **2**, and **3**, with hexamethylcyclotrisiloxane as the internal standard. The μ_{eff} found for complexes **1** and **2** are not within the range of Ce(III) complex magnetic moments whereas complex **3** is well within the range.[3]

Magnetism of complexes 1–3:

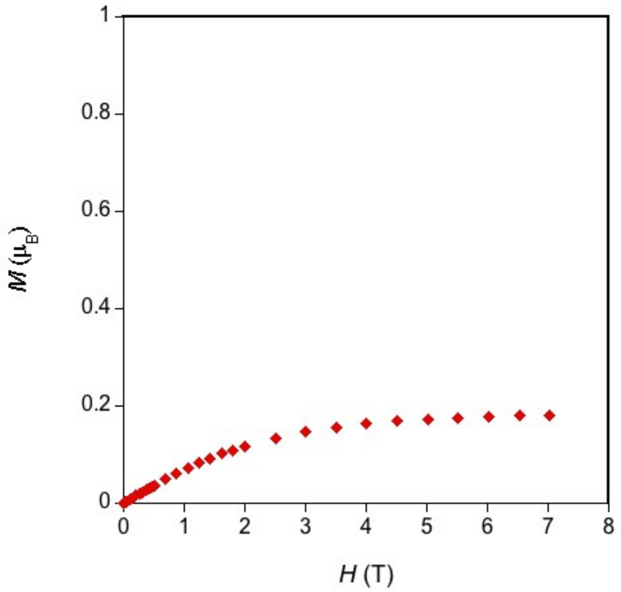

Figure S5. Field dependence of complex $Li_4(py)_4[Ce(PhNNPh)_4]$ at 2 K.

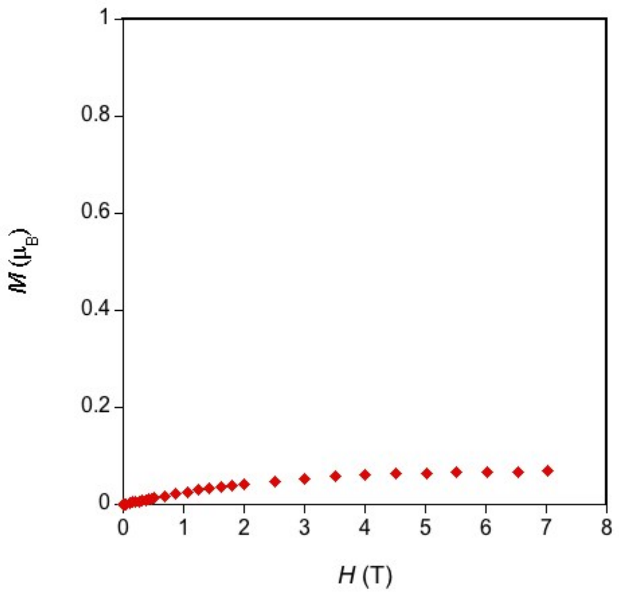

Figure S6. Field dependence of complex $Na_4(py)_8[Ce(PhNNPh)_4]$ at 2 K.

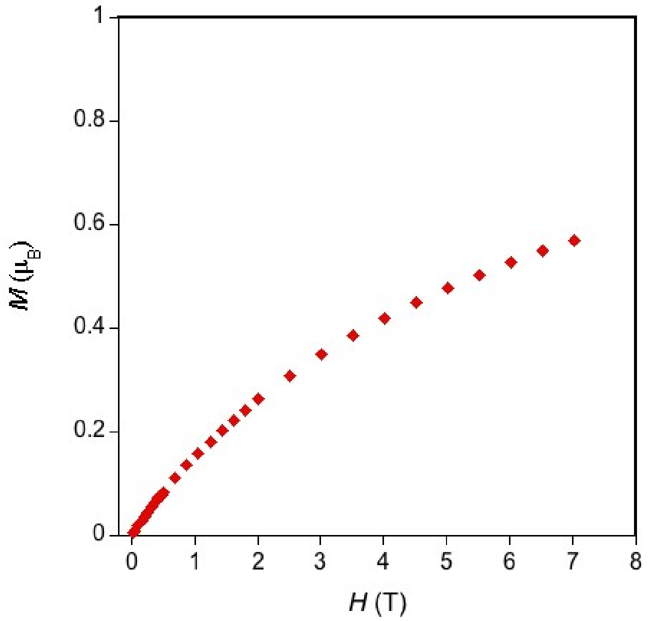

Figure S7. Field dependence of complex $K_5(py)_7[Ce(PhNNPh)_4]$ at 2 K.

Ce L_{III}-edge XAS Spectroscopy

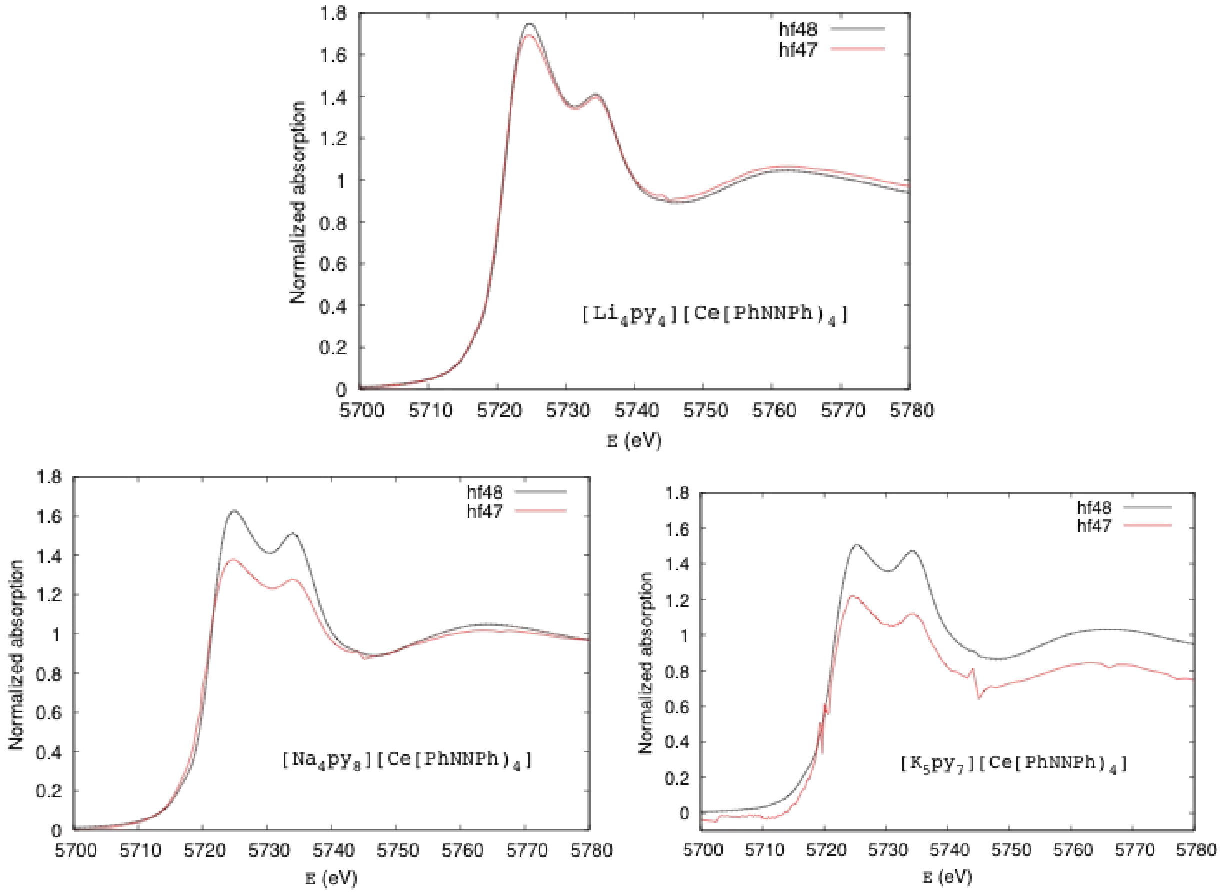

Figure S8. Normalized absorption (A) as a function of the incident X-ray energy (E) in the Ce L_{III} near-edge region at T = 30 K. The red and black traces represent two different measurements.

NMR Spectra of Complexes 1-3:

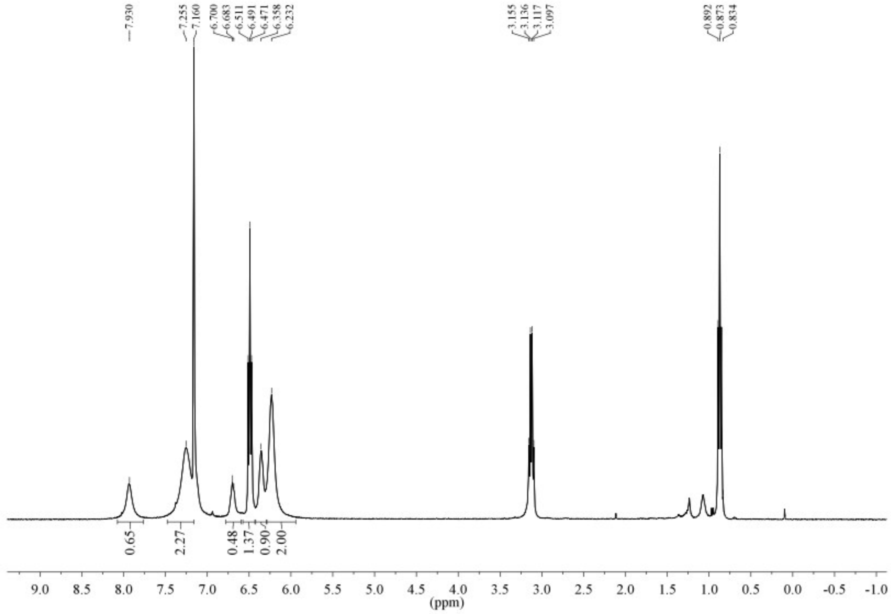

Figure S9. ^{1}H NMR spectrum of $Li_4(py)_4[Ce(PhNNPh)_4]$ collected in C_6D_6.

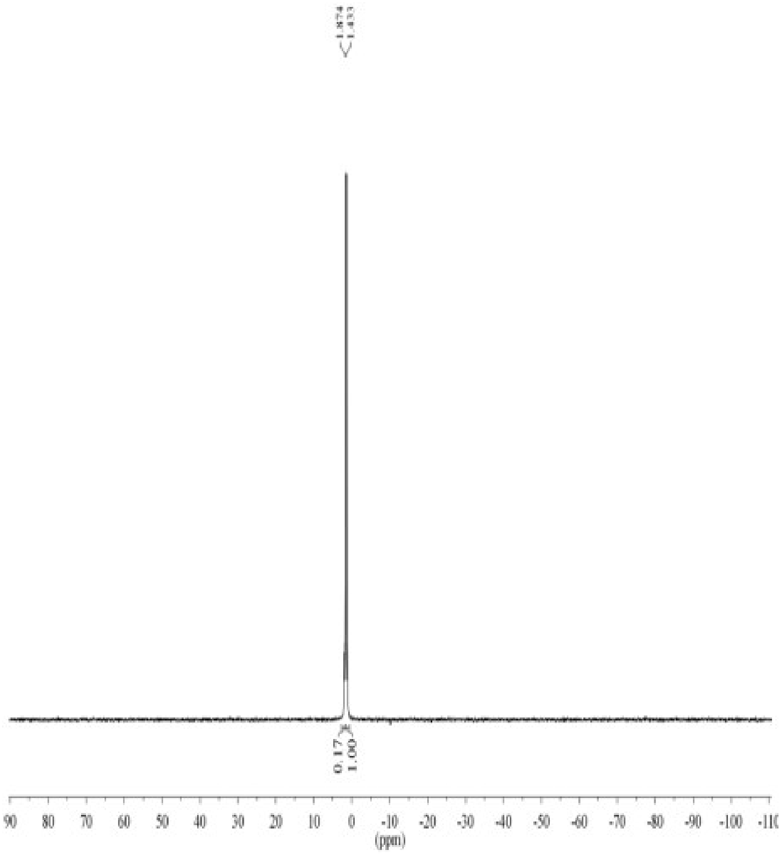

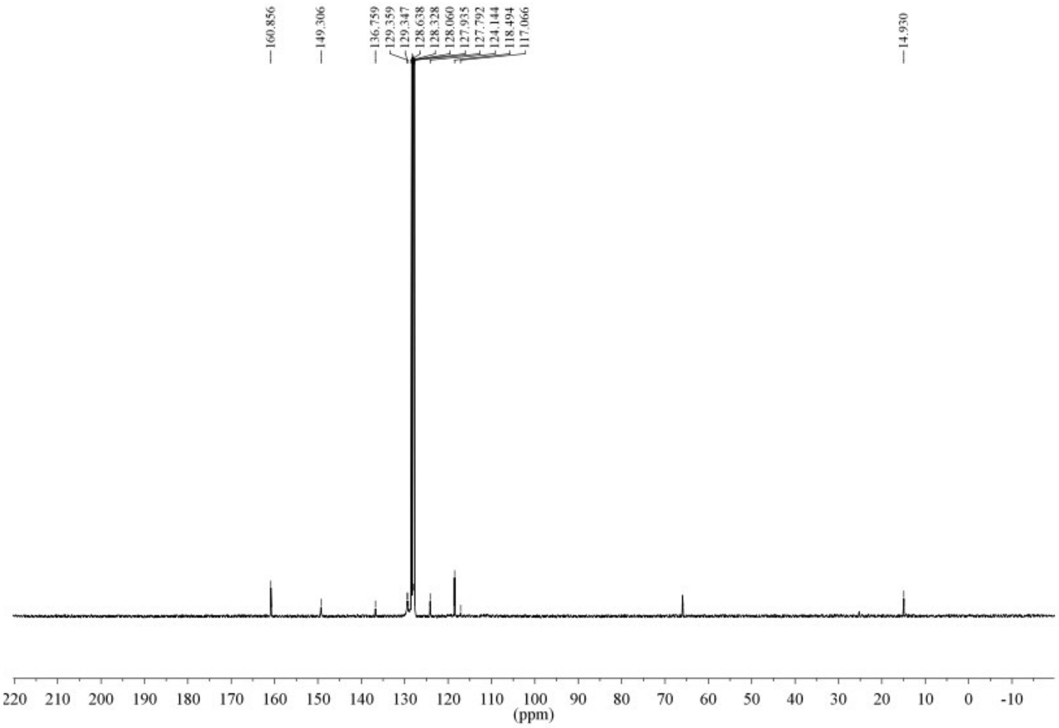

Figure S10. ^{7}Li NMR (top) and ^{13}C NMR (bottom) spectra of $Li_4(py)_4[Ce(PhNNPh)_4]$ collected in C_6D_6.

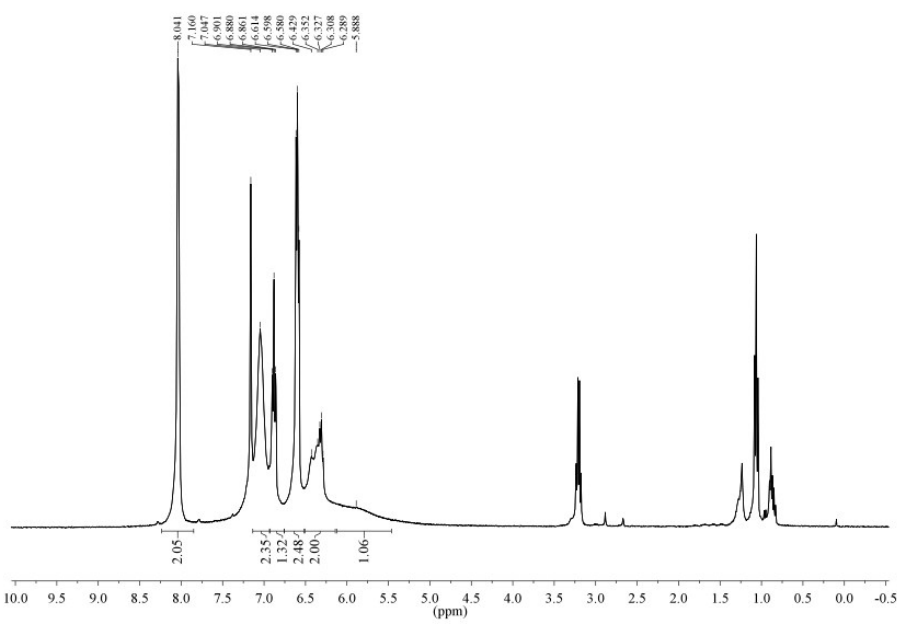

Figure S11. ^{1}H NMR spectrum of $Na_4(py)_8[Ce(PhNNPh)_4]$ collected in C_6D_6.

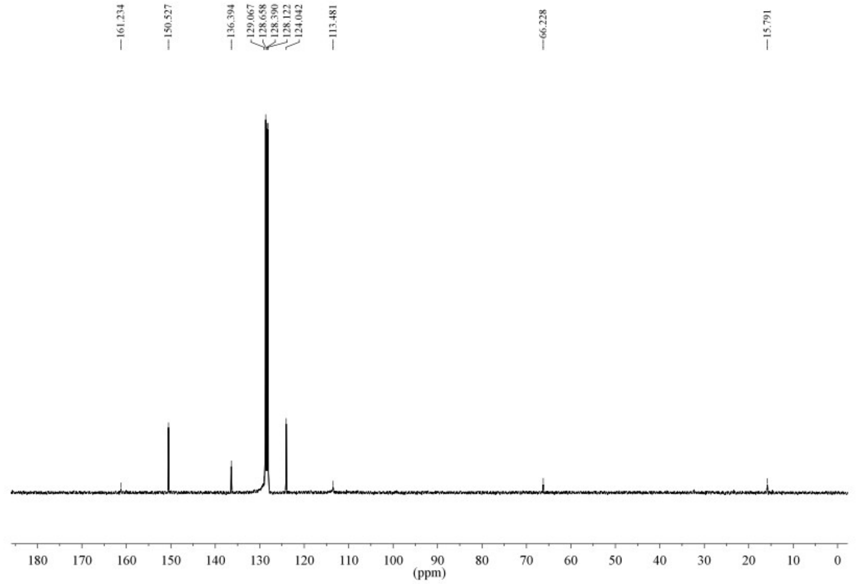

Figure S12. ^{13}C NMR spectrum of $Na_4(py)_4[Ce(PhNNPh)_4]$ collected in C_6D_6.

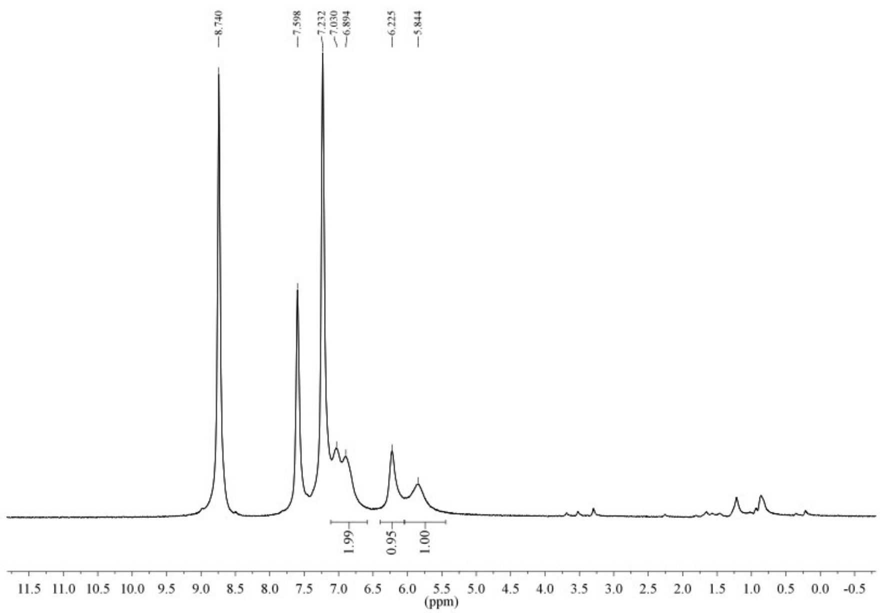

Figure S13. ^{1}H NMR spectrum of $K_5(py)_7[Ce(PhNNPh)_4]$ collected in pyridine-d_5.

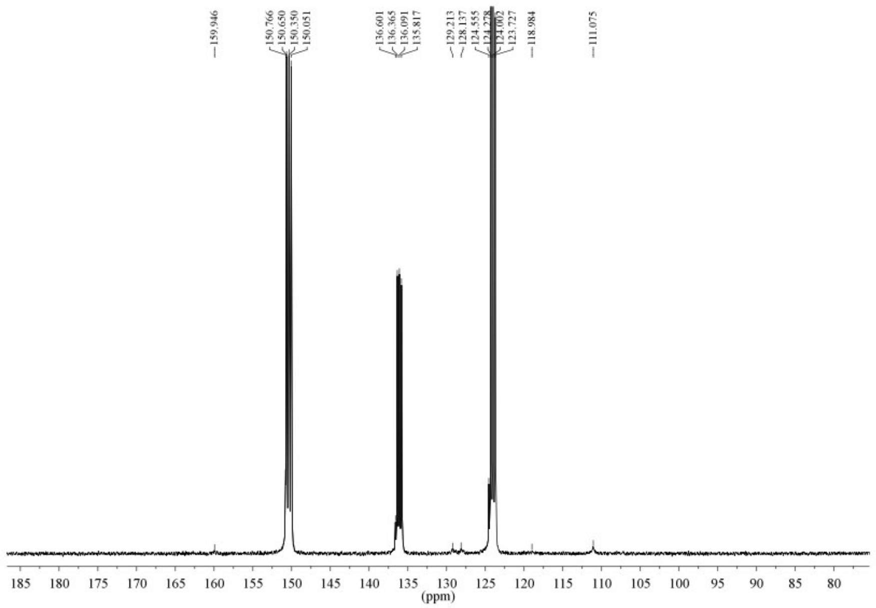

Figure S14. ^{13}C NMR spectrum of $K_5(py)_7[Ce(PhNNPh)_4]$ collected in pyridine-d_5.

^{1}H NMR Spectra from Metathesis Reactions

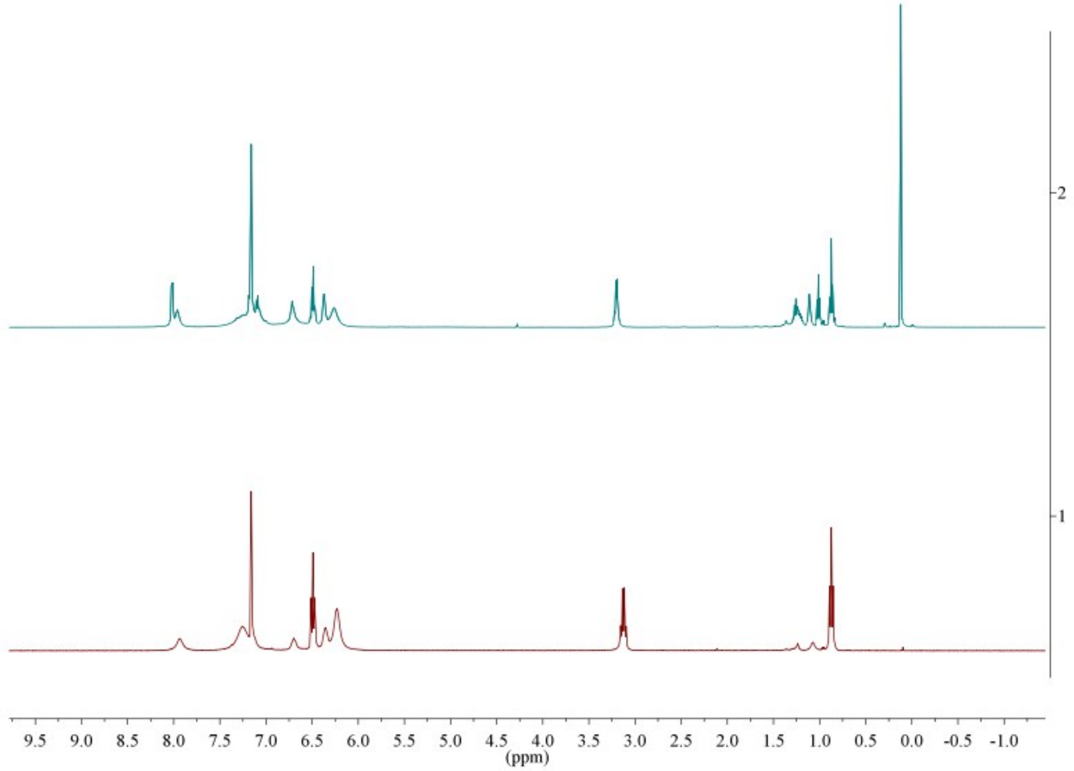

Figure S15. ^{1}H NMR spectrum of the metathesis reaction of complex **3** with 4 equiv. of LiI in C_6D_6, where 2.0 µL of TMS_2O was used as an internal standard to determine percent conversion (top). To compare the products, the ^{1}H NMR spectrum of crystals of complex **1** in C_6D_6 was included (bottom).

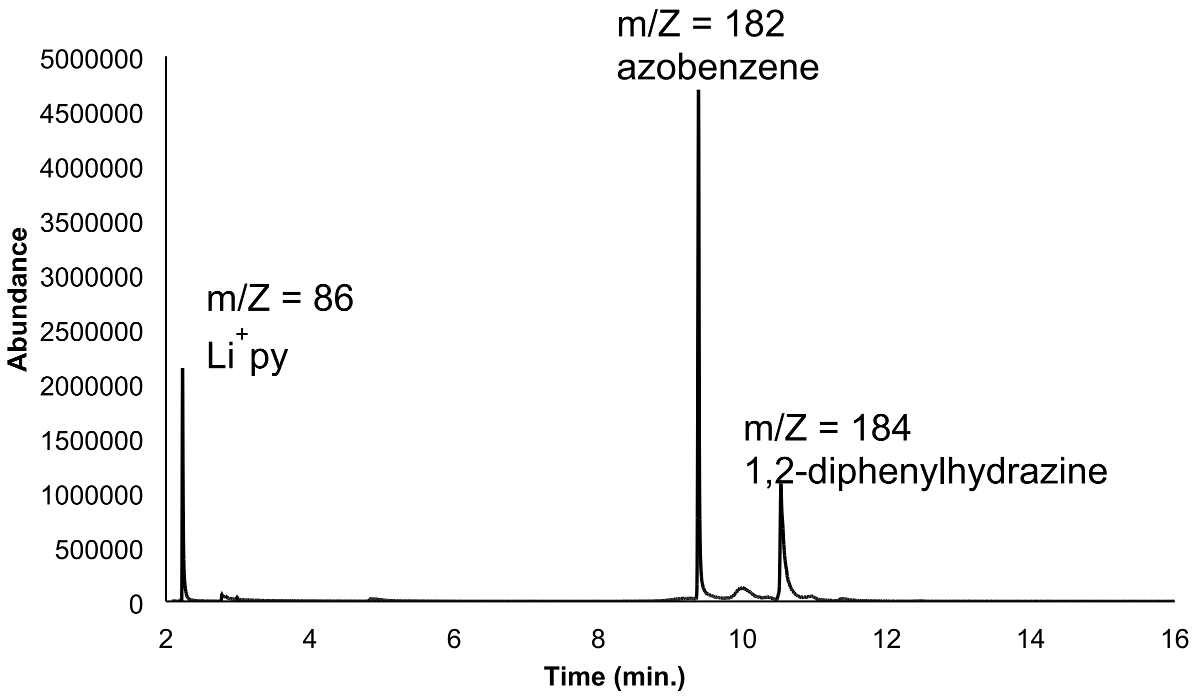

Figure S16. Gas chromatogram of the metathesis reaction of complex **3** with 4 equiv. of LiI. Complex **3** was reacted with LiI first in diethyl ether. The reaction was then acidified with $HNEt_3Cl$, filtered over celite, and run on the GC/MS. At 2.233 min, m/Z = 86, corresponds to pyridine with Li^+; the next trace at 9.352 min, m/Z = 182, corresponds to azobenzene; the final trace that can be integrated at 10.54 min, m/Z =184, corresponds to 1,2-diphenylhydrazine.

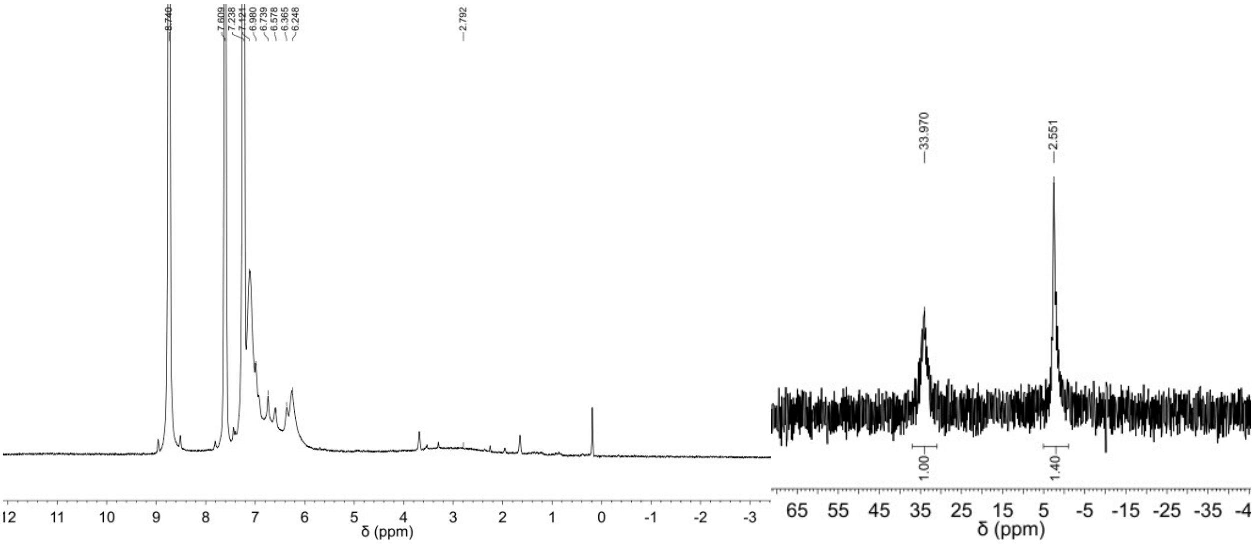

Figure S17. ^{1}H (left) and ^{7}Li (right) NMR spectra of the metathesis reaction of complex **1** with 5 equiv. of KI in pyridine-d_5.

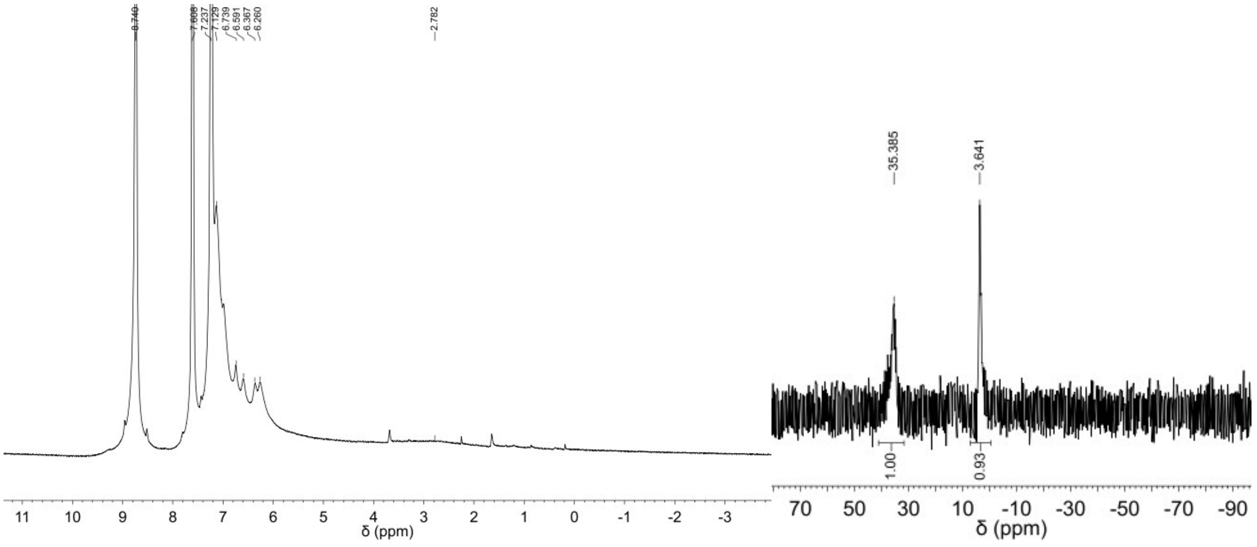

Figure S18. ^{1}H (left) and ^{7}Li (right) NMR spectra of the metathesis reaction of complex **1** with 5 equiv. of KI and 0.5 equiv. of PhNHNHPh in pyridine-d_5.

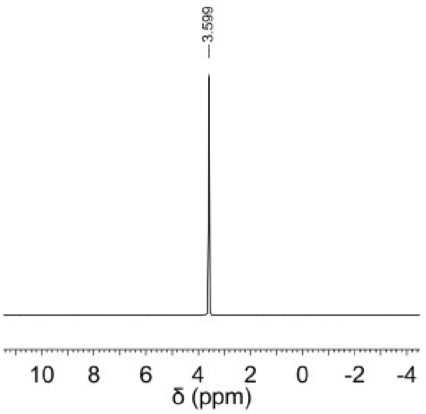

Figure S19. ^{7}Li NMR spectrum of LiI in pyridine-d_5.

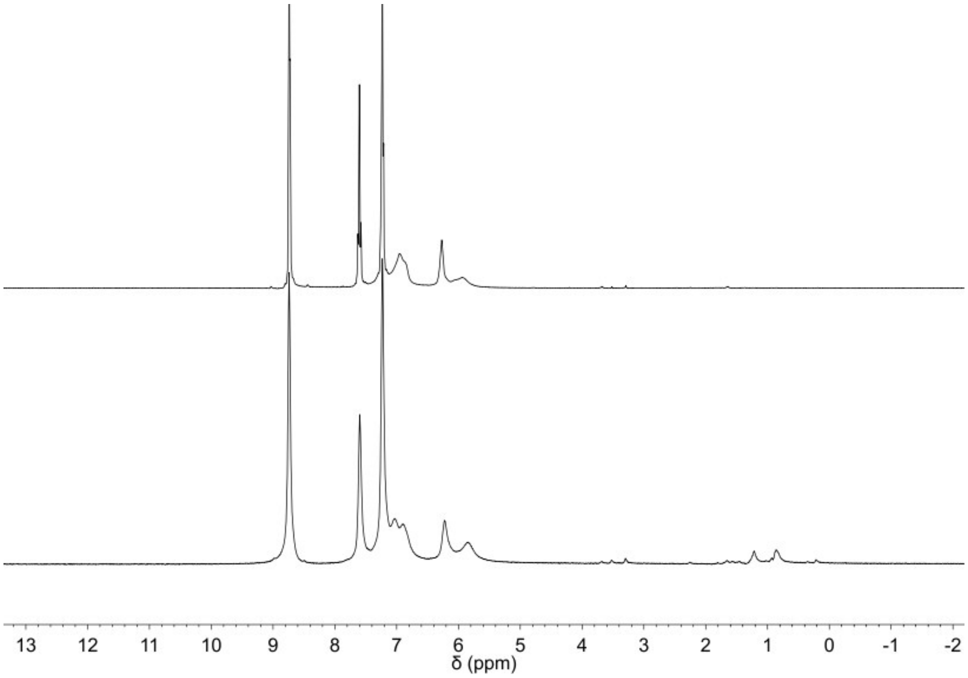

Figure S20. ^1H NMR spectrum of the metathesis reaction of complex **2** with 5 equiv. of KI and in pyridine-d_5 (top). To compare products, the ^1H NMR spectrum of pure complex **3** in pyridine-d_5 was provided at bottom.

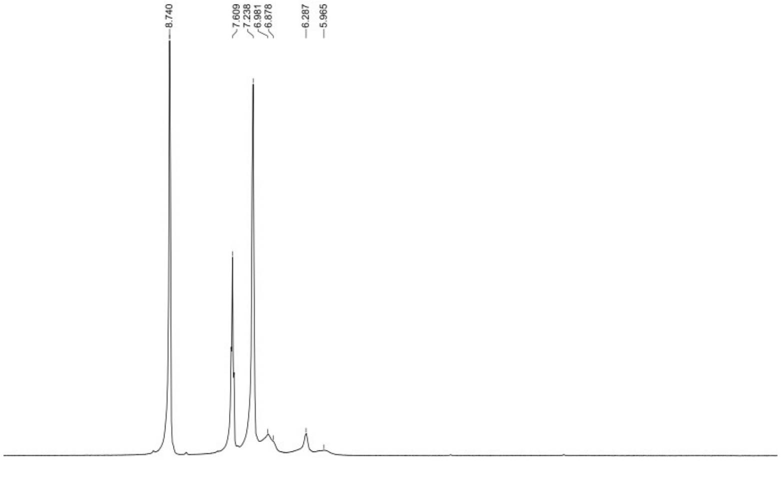

Figure S21. ^1H NMR spectrum of the metathesis reaction of complex **2** with 5 equiv. of KI and 0.5 equiv. of PhNHNHPh in pyridine-d_5.

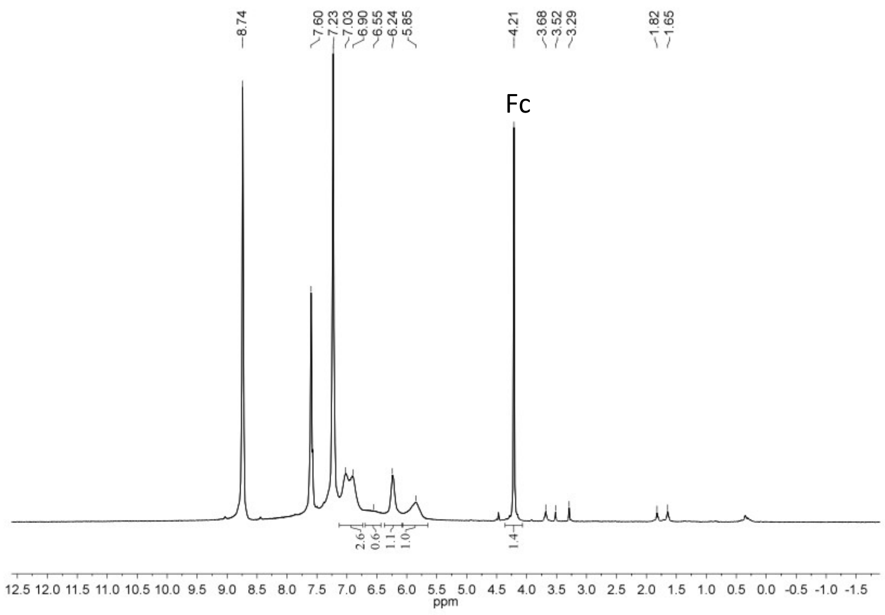

Figure S22. ^{1}H NMR spectrum in pyridine-d_5 of the following crude reaction mix: $Ce\{N(SiHMe_2)_2\}_4$ was prepared in situ by reacting $K[Ce\{N(SiHMe_2)_2\}_4]$ with FcOTf.[4] In a diethyl ether solution, KH and 1,2-diphenylhydrazine were added to $Ce\{N(SiHMe_2)_2\}_4$. The reaction resulted in complex **3** and ferrocene.

FTIR Spectra of Complexes 1-3:

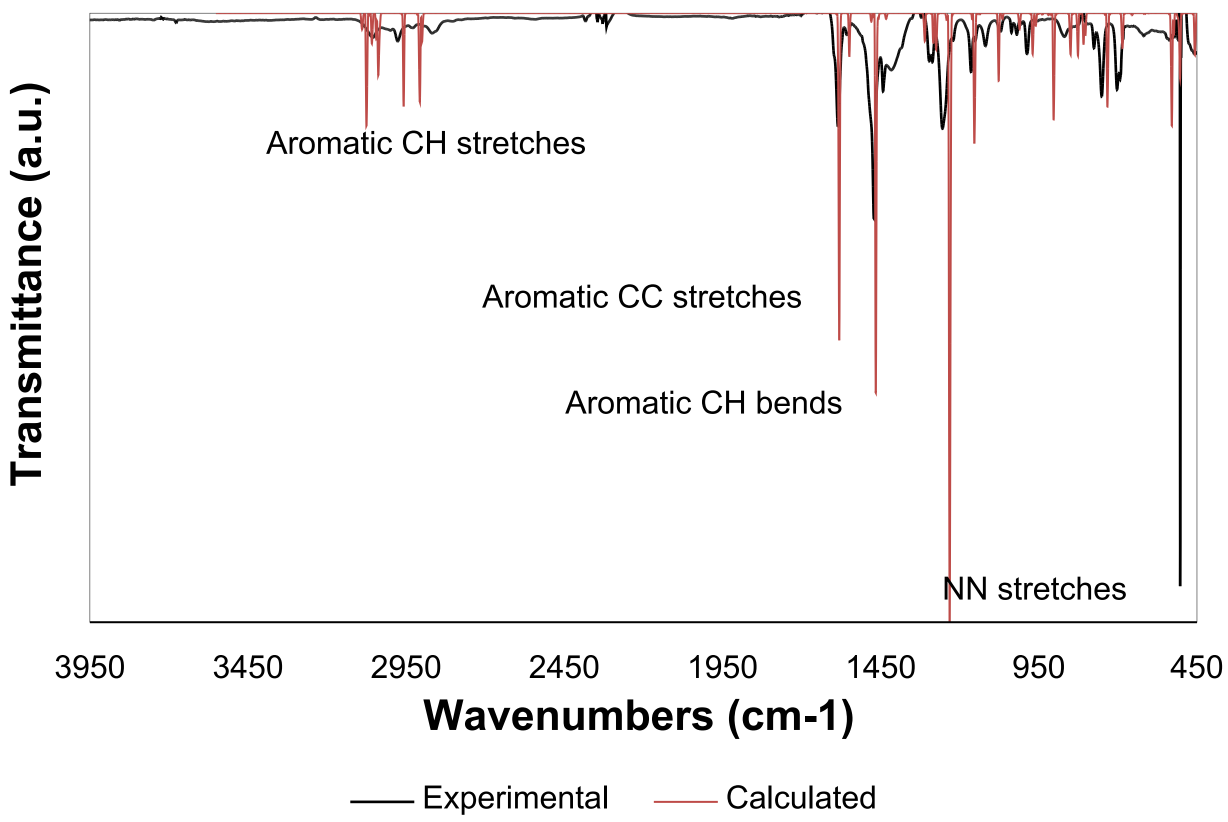

Figure S23. Experimental (black) FTIR spectrum of $Li_4(py)_4[Ce(PhNNPh)_4]$ collected in C_6D_6 and its calculated spectrum (red) of $Li_4(OMe_2)_4[Ce(PhNNPh)_4]$, where the calculated energies are scaled by 0.9594.[5]

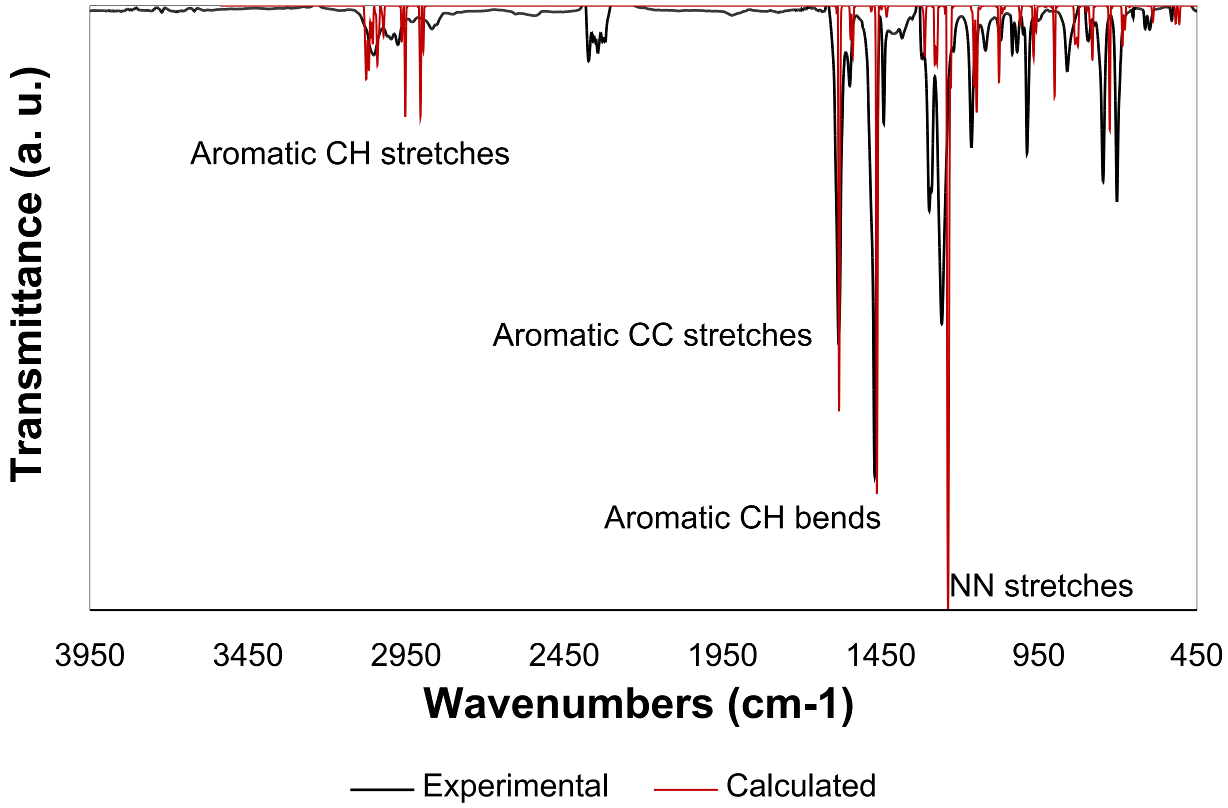

Figure S24. Experimental (black) FTIR spectrum of $Na_4(py)_8[Ce(PhNNPh)_4]$ collected in C_6D_6 and its calculated spectrum (red) of $Na_4(OMe_2)_4[Ce(PhNNPh)_4]$, where the calculated energies are scaled by 0.9594.[5]

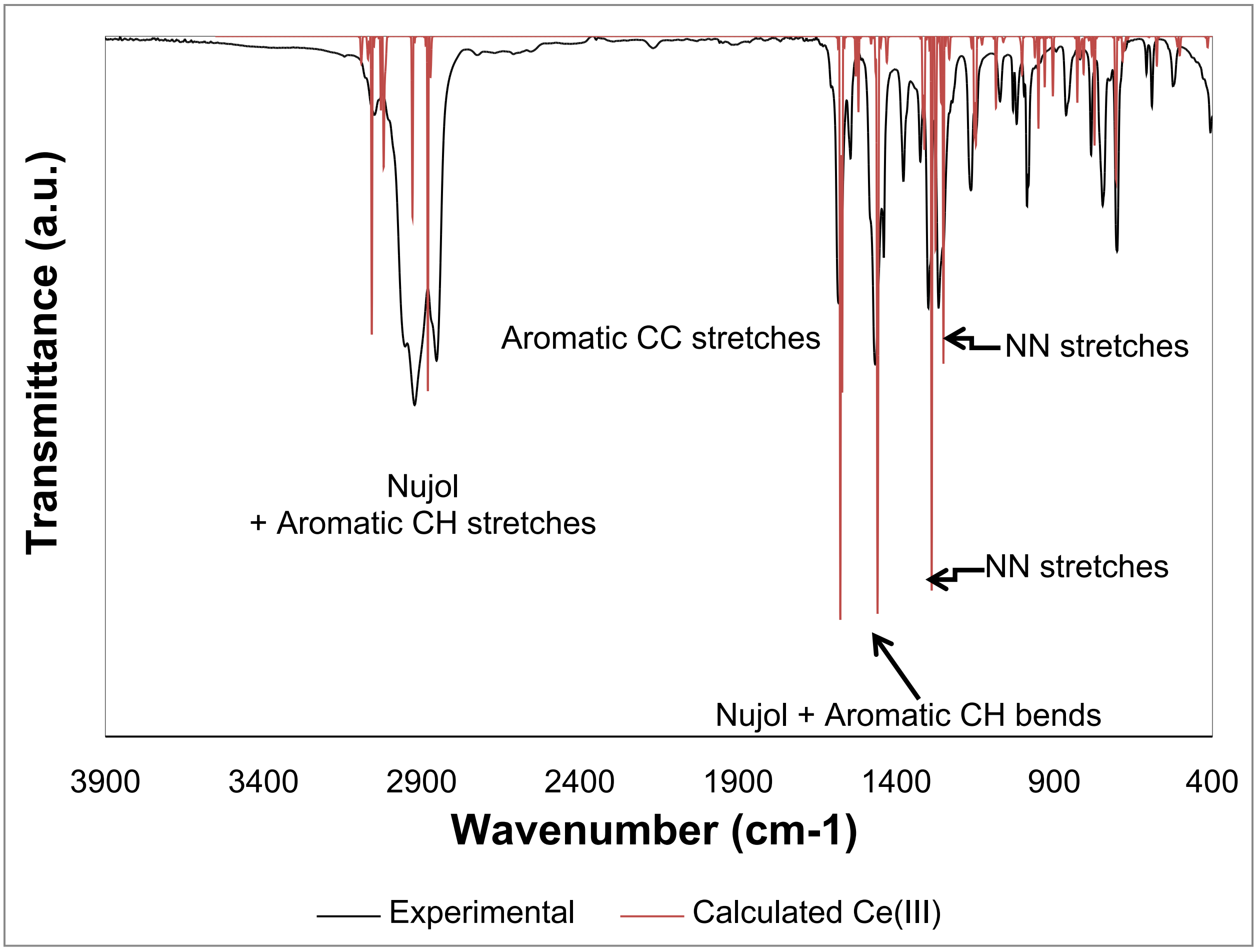

Figure S25. Experimental (black) FTIR spectrum of $K_5(py)_7[Ce(PhNNPh)_4]$ collected in nujol and its calculated spectrum (red) of $K_4(OMe_2)_4[Ce(PhNNPh)_4]^-$, where the calculated energies are scaled by 0.9594.[5]

UV-Vis Absorption Spectra of 1-3:

UV-Vis absorption spectra of **1** and **2** show broad ligand-to-metal charge transfer bands centered at 18,315 cm^{-1} for complex **1** and 19,666 cm^{-1} for complex **2** measured in fluorobenzene, which are characteristic of Ce(IV) complexes.[6] Because **3** is insoluble in non-coordinating solvents and **1** and **2** are unstable in coordinating solvents, **3** was experimentally inaccessible for comparison in the same solvent.

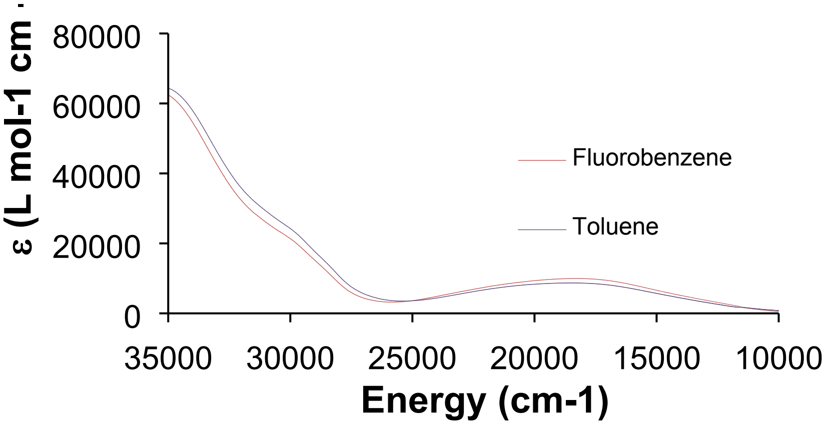

Figure S26. UV-Vis spectra of **1** in fluorobenzene (red) and toluene (blue).

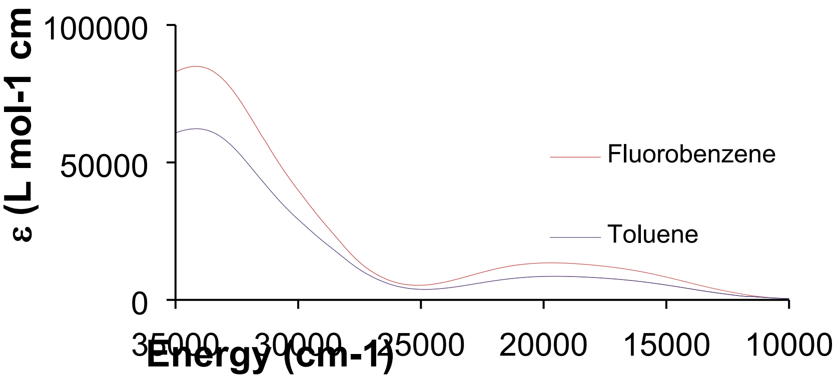

Figure S27. UV-Vis spectra of **2** in fluorobenzene (red) and toluene (blue).

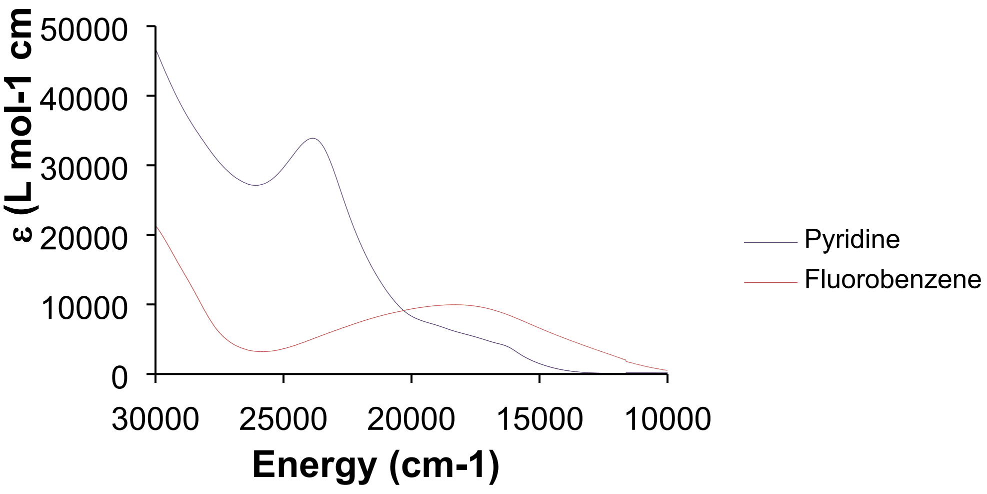

Figure S28. UV-Vis Spectra of $Li_4(py)_4[Ce(PhNNPh)_4]$ collected in pyridine and fluorobenzene.

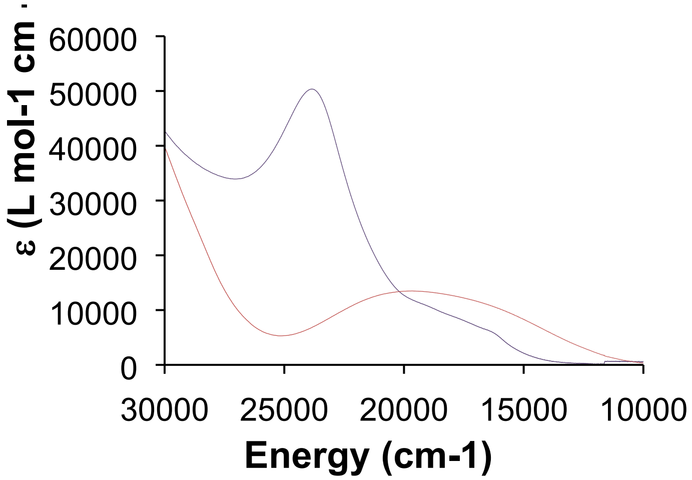

Figure S29. UV-Vis Spectra of $Na_4(py)_8[Ce(PhNNPh)_4]$ collected in pyridine and fluorobenzene.

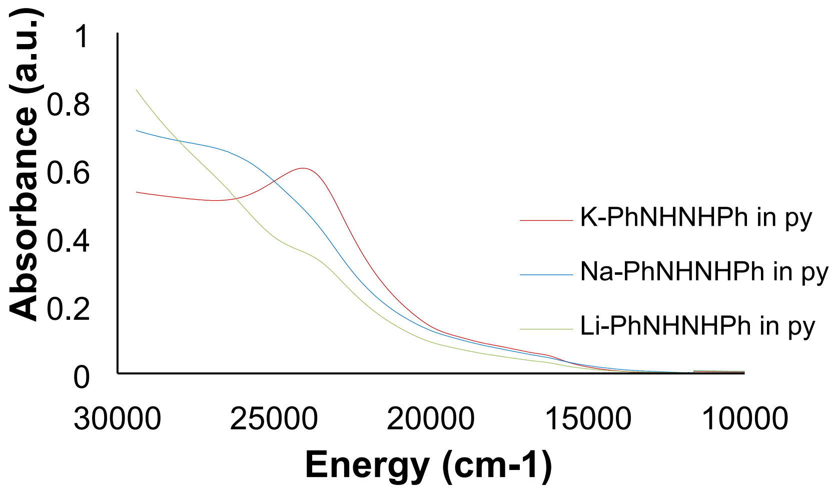

Figure S30. UV-Vis spectra of 1,2-diphenylhydrazine deprotonated with $KN(SiMe_3)_2$ (red), $NaN(SiMe_3)_2$ (blue) or $LiN(SiMe_3)_2$ (green) collected in pyridine.

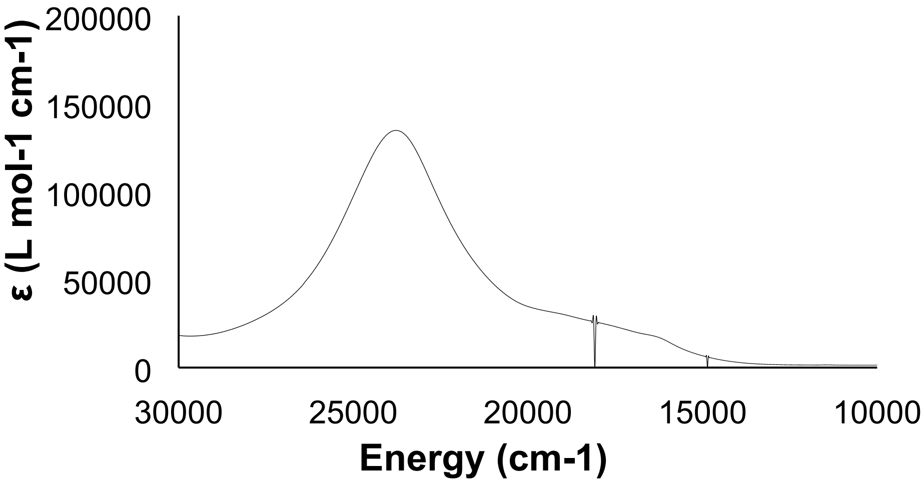

Figure S31. UV-Vis spectrum of $K_5(py)_7[Ce(PhNNPh)_4]$ in pyridine.

Electrochemistry of 1-2 in fluorobenzene:

To quantify how much the 1,2-diphenylhydrazide stabilized the Ce(IV) oxidation state, cyclic voltammetry was measured. The metal redox of both complexes **1** and **2** centers on ~ –1.9 V vs Fc/Fc^+ (Figures S26–S29). For the CVs of complexes **1** and **2**, the scans begin at the open circuit potential and then the scans sweep to more reducing potentials. The electrochemistry of complex **3** in fluorobenzene was unreliable as complex **3** is extremely sensitive to solvent conditions and it is effectively insoluble in fluorobenzene.

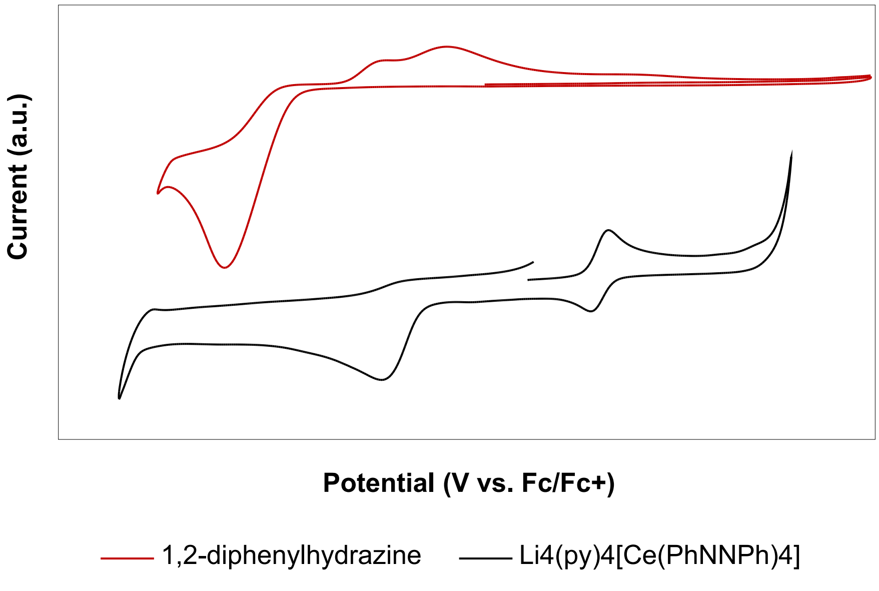

Figure S32. Cyclic voltammogram of 1,2-diphenylhydrazine (top, red) and $Li_4(py)_4[Ce(PhNNPh)_4]$ (bottom, black) in a solution of $[NBu_4][BAr^F_4]$ in fluorobenzene, v = 100 mV/s.

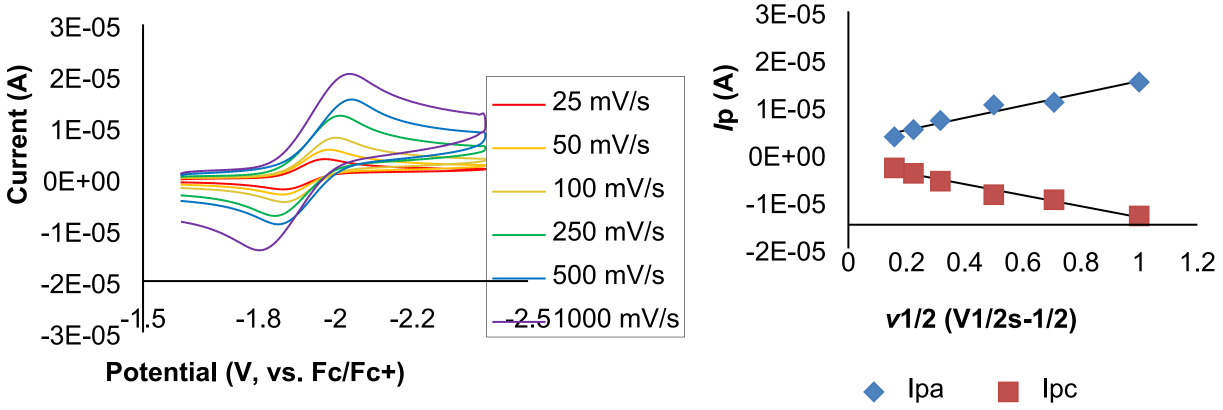

Figure S33. Isolation scans of $Li_4(py)_4[Ce(PhNNPh)_4]$ in a solution of $[NBu_4][BAr^F_4]$ in fluorobenzene at varying scan rates (left). At right, i_p vs. $v^{1/2}$ plot.

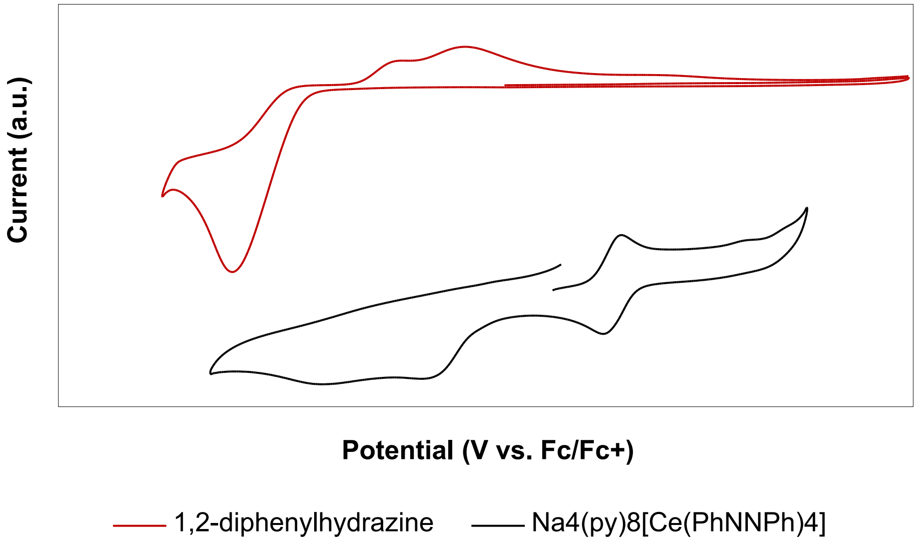

Figure S34. Cyclic voltammogram of 1,2-diphenylhydrazine (top, red) and $Na_4(py)_8[Ce(PhNNPh)_4]$ (bottom, black) in a solution of $[NBu_4][BAr^F_4]$ in fluorobenzene, v = 100 mV/s.

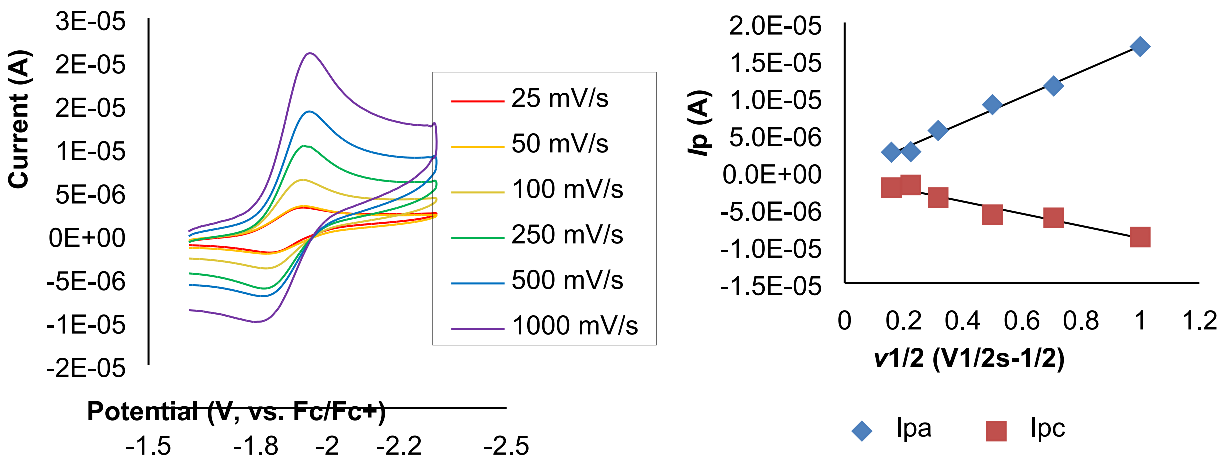

Figure S35. Isolation scans of $Na_4(py)_8[Ce(PhNNPh)_4]$ in a solution of $[NBu_4][BAr^F{}_4]$ in fluorobenzene at varying scan rates(left). At right, i_p vs. $v^{1/2}$ plot.

Electrochemistry of 3 in THF:

Because the complex **3** was insoluble and unstable in fluorobenzene, the complex was measured instead in THF. For the CV of complex **3**, the scan begins at the open circuit potential and then the scans sweep to more oxidizing potentials.

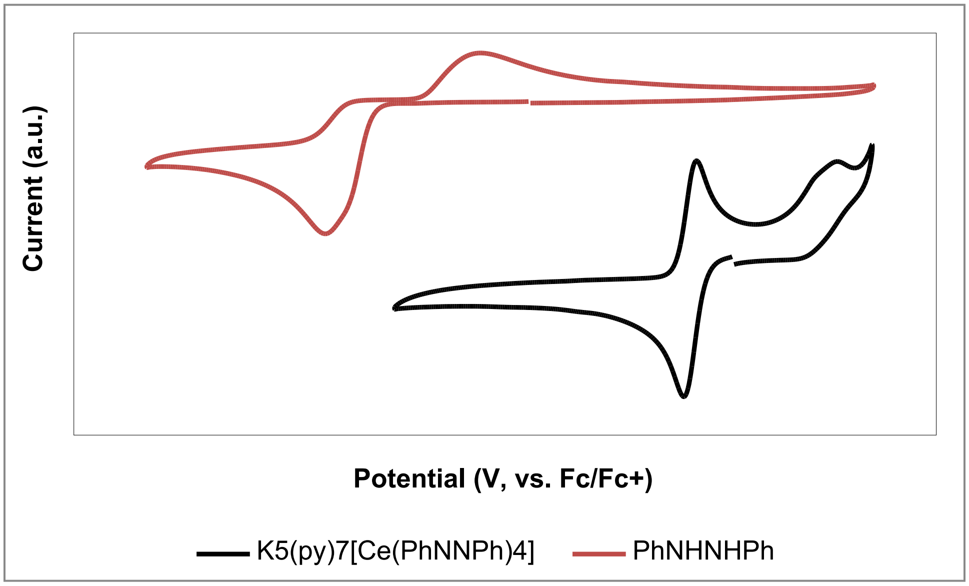

Figure S36. Cyclic voltammogram of 1,2-diphenylhydrazine (top, red) and $K_5(py)_7[Ce(PhNNPh)_4]$ (bottom, black) in a solution of $[NPr_4][BAr^F{}_4]$ in THF, $v = 100$ mV/s.

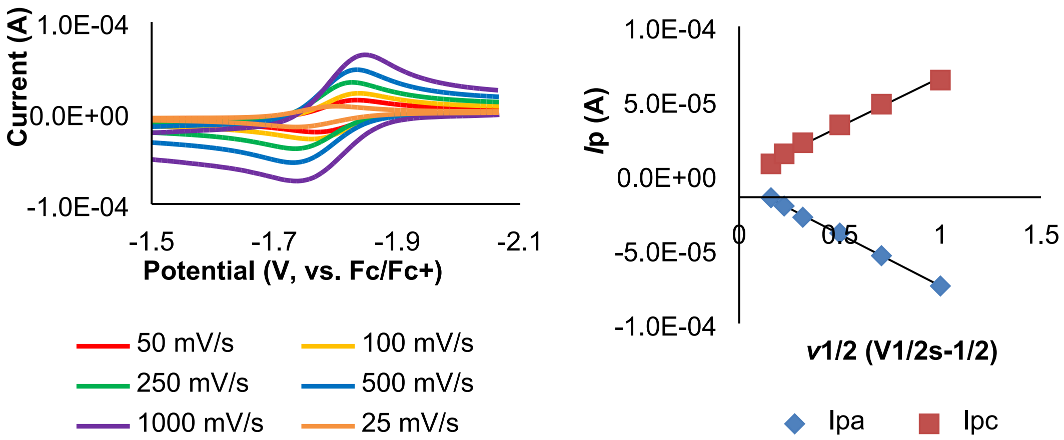

Figure S37. Isolation scans of $K_5(py)_7[Ce(PhNNPh)_4]$ in a solution of $[NPr_4][BAr^F{}_4]$ in THF at varying scan rates (left). At right, i_p vs. $v^{1/2}$ plot.

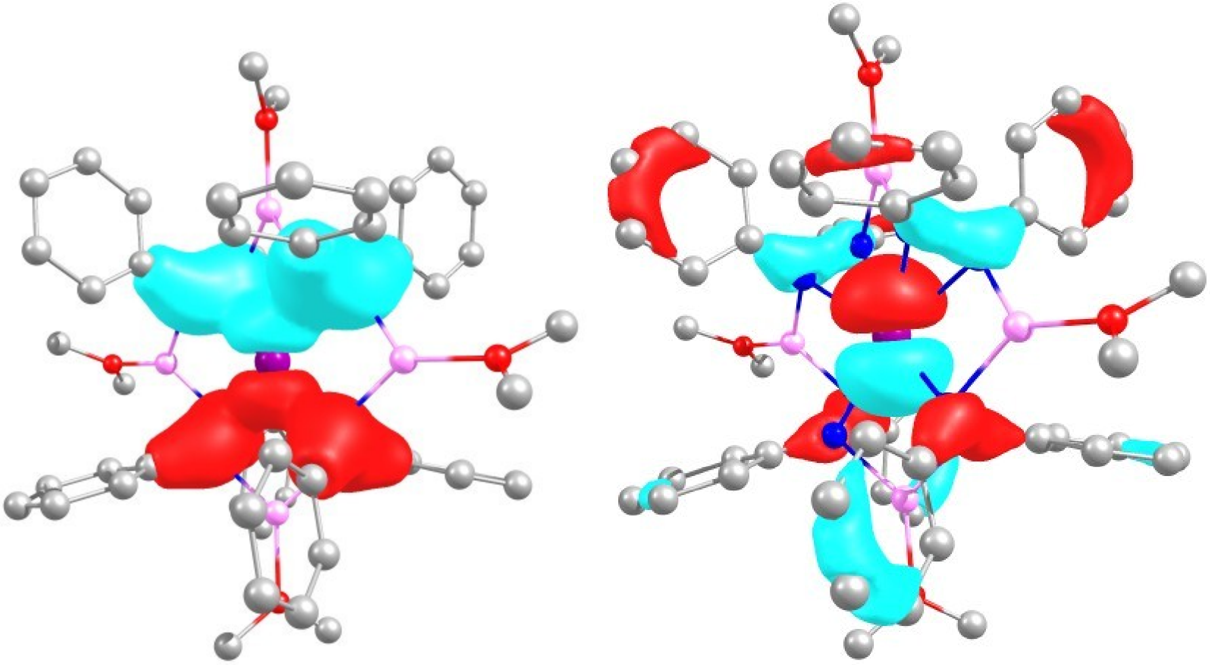

Figure S38. Atomic orbitals 87 and 101 of the calculated $Li_4(OMe)_4[Ce(PhNNPh)_4]$ complex. Ce^{4+} p orbitals interact with N–N σ bonds.

<u>Optimized Coordinates for Ce(IV) Calculations:</u>

Table S4. $Li_4(OMe_2)_4[Ce(PhNNPh)_4]$ (1-OMe$_2$)

Ce	0.000000000	0.000000000	0.000000000
O	-5.235507867	0.000000001	0.000000000

O	0.000000001	5.235496008	0.000000000
N	-1.964886567	1.368167716	0.583874073
N	-1.368126731	1.964875015	-0.583971161
C	-2.228480291	2.200709936	1.668705336
C	-2.399592432	3.601175266	1.571997585
H	-2.336271347	4.075837702	0.598868766
C	-2.702787622	4.365647544	2.702379372
H	-2.842173999	5.439563282	2.591771151
C	-2.855189833	3.772061994	3.955568101
H	-3.093964847	4.371832562	4.829137581
C	-2.707949063	2.382265517	4.061015532
H	-2.827361063	1.895417913	5.026407854
C	-2.401902163	1.607547824	2.946900045
H	-2.284607211	0.530323833	3.045601465
C	-2.200604805	2.228350404	-1.668881467
C	-1.607355827	2.401721619	-2.947042160
H	-0.530115512	2.284497481	-3.045651213
C	-2.382008496	2.707632848	-4.061239924
H	-1.895094739	2.827009023	-5.026603321
C	-3.771823181	2.854785468	-3.955910452
H	-4.371542864	3.093454609	-4.829543778
C	-4.365494553	2.702436576	-2.702756157
H	-5.439428289	2.841757552	-2.592240991
C	-3.601088328	2.399376628	-1.572293495
H	-4.075823102	2.336095734	-0.599197416
C	-6.015086933	0.557059699	1.053352937
H	-5.320434370	0.963090348	1.791348376
H	-6.637862566	-0.215542854	1.523516852
C	0.556943052	6.015073915	1.053414253
H	-0.215714812	6.637835419	1.523505570
H	1.361426612	6.659215821	0.672690960
Li	-3.282905430	0.000000000	0.000000000
Li	0.000000000	3.282898297	0.000000000
C	-6.015086933	-0.557059694	-1.053352937
H	-5.320434370	-0.963090343	-1.791348376
H	-6.637862566	0.215542856	-1.523516852
C	-0.556943047	6.015073915	-1.053414253
H	0.215714813	6.637835419	-1.523505570
H	-1.361426607	6.659215821	-0.672690960
O	-0.000000001	-5.235496008	0.000000000

N	-1.964886567	-1.368167716	-0.583874073
N	-1.368126731	-1.964875015	0.583971161
C	-2.228480296	-2.200709936	-1.668705336
C	-2.399592432	-3.601175266	-1.571997585
H	-2.336271347	-4.075837702	-0.598868766
C	-2.702787622	-4.365647544	-2.702379372
H	-2.842174004	-5.439563229	-2.591771151
C	-2.855189833	-3.772061994	-3.955568101
H	-3.093964847	-4.371832562	-4.829137581
C	-2.707949063	-2.382265517	-4.061015532
H	-2.827361063	-1.895417913	-5.026407854
C	-2.401902163	-1.607547824	-2.946900045
H	-2.284607211	-0.530323833	-3.045601465
C	-2.200604805	-2.228350404	1.668881467
C	-1.607355827	-2.401721619	2.947042160
H	-0.530115512	-2.284497481	3.045651213
C	-2.382008496	-2.707632848	4.061239924
H	-1.895094739	-2.827009023	5.026603321
C	-3.771823181	-2.854785468	3.955910452
H	-4.371542864	-3.093454609	4.829543778
C	-4.365494553	-2.702436576	2.702756157
H	-5.439428289	-2.841757552	2.592240991
C	-3.601088333	-2.399376628	1.572293495
H	-4.075823102	-2.336095734	0.599197416
C	0.556943047	-6.015073915	-1.053414253
H	-0.215714813	-6.637835419	-1.523505570
H	1.361426607	-6.659215821	-0.672690960
Li	0.000000000	-3.282898297	0.000000000
C	-0.556943052	-6.015073915	1.053414253
H	0.215714812	-6.637835419	1.523505570
H	-1.361426612	-6.659215821	0.672690960
O	5.235507867	-0.000000001	0.000000000
N	1.964886567	-1.368167716	0.583874073
N	1.368126731	-1.964875015	-0.583971161
C	2.228480291	-2.200709936	1.668705336
C	2.399592432	-3.601175266	1.571997585
H	2.336271347	-4.075837702	0.598868766
C	2.702787622	-4.365647544	2.702379372
H	2.842173999	-5.439563282	2.591771151
C	2.855189833	-3.772061994	3.955568101

H	3.093964847	-4.371832562	4.829137581
C	2.707949063	-2.382265517	4.061015532
H	2.827361063	-1.895417913	5.026407854
C	2.401902163	-1.607547824	2.946900045
H	2.284607211	-0.530323833	3.045601465
C	2.200604805	-2.228350404	-1.668881467
C	1.607355827	-2.401721619	-2.947042160
H	0.530115512	-2.284497481	-3.045651213
C	2.382008496	-2.707632848	-4.061239924
H	1.895094739	-2.827009023	-5.026603321
C	3.771823181	-2.854785468	-3.955910452
H	4.371542864	-3.093454609	-4.829543778
C	4.365494553	-2.702436576	-2.702756157
H	5.439428289	-2.841757552	-2.592240991
C	3.601088328	-2.399376628	-1.572293495
H	4.075823102	-2.336095734	-0.599197416
C	6.015086933	-0.557059699	1.053352937
H	5.320434370	-0.963090348	1.791348376
H	6.637862566	0.215542854	1.523516852
Li	3.282905430	0.000000000	0.000000000
C	6.015086933	0.557059694	-1.053352937
H	5.320434370	0.963090343	-1.791348376
H	6.637862566	-0.215542856	-1.523516852
N	1.964886567	1.368167716	-0.583874073
N	1.368126731	1.964875015	0.583971161
C	2.228480296	2.200709936	-1.668705336
C	2.399592432	3.601175266	-1.571997585
H	2.336271347	4.075837702	-0.598868766
C	2.702787622	4.365647544	-2.702379372
H	2.842174004	5.439563229	-2.591771151
C	2.855189833	3.772061994	-3.955568101
H	3.093964847	4.371832562	-4.829137581
C	2.707949063	2.382265517	-4.061015532
H	2.827361063	1.895417913	-5.026407854
C	2.401902163	1.607547824	-2.946900045
H	2.284607211	0.530323833	-3.045601465
C	2.200604805	2.228350404	1.668881467
C	1.607355827	2.401721619	2.947042160
H	0.530115512	2.284497481	3.045651213
C	2.382008496	2.707632848	4.061239924

H	1.895094739	2.827009023	5.026603321
C	3.771823181	2.854785468	3.955910452
H	4.371542864	3.093454609	4.829543778
C	4.365494553	2.702436576	2.702756157
H	5.439428289	2.841757552	2.592240991
C	3.601088333	2.399376628	1.572293495
H	4.075823102	2.336095734	0.599197416
H	-0.962906914	-5.320420717	1.791445951
H	0.962906914	-5.320420717	-1.791445951
H	-6.659216773	-1.361511614	-0.672542484
H	-6.659216773	1.361511614	0.672542484
H	-0.962906914	5.320420717	-1.791445951
H	0.962906914	5.320420717	1.791445951
H	6.659216773	-1.361511614	0.672542484
H	6.659216773	1.361511614	-0.672542484

Lowest Energy Frequencies (cm^{-1}) –16.32, –12.12, –12.08, –11.37, 9.20, 20.57

Sum of Electronic and Thermal Free Energies (Hartrees) –3415.92

Table S5. $Na_4(OMe_2)_4[Ce(PhNNPh)_4]$ (2-OMe_2)

Ce	0.000000361	-0.000001339	-0.000019559
Na	-2.612491927	2.110812395	-1.058384767
Na	2.110752117	2.612446429	1.058448760
Na	2.612490409	-2.110827572	-1.058362949
Na	-2.110753392	-2.612451424	1.058451417
O	-4.039218721	2.667653316	-2.776750233
O	2.667557720	4.039087400	2.776883650
O	4.039235554	-2.667646791	-2.776719906
O	-2.667555069	-4.039085310	2.776893783
N	-0.245509391	1.997500195	-1.377494899
N	0.001331277	2.442631227	-0.029507024
N	1.997516239	0.245290331	1.377384804
N	2.442633222	-0.001348832	0.029368073
N	0.245508530	-1.997506894	-1.377488496
N	-0.001330441	-2.442635942	-0.029499454
N	-1.997513773	-0.245293049	1.377385502
N	-2.442633116	0.001349278	0.029370692

C	0.541120626	2.538931273	-2.383815546
C	1.385548067	3.664569679	-2.231058590
H	1.431247428	4.165115629	-1.268668434
C	2.120358499	4.163083716	-3.308093098
H	2.756827446	5.032293654	-3.154392905
C	2.041513237	3.576281885	-4.572016473
H	2.612397146	3.973857001	-5.406316663
C	1.195061905	2.472610362	-4.744262282
H	1.107452637	2.003645128	-5.722643159
C	0.457724998	1.964829204	-3.680213148
H	-0.198582205	1.109704493	-3.824003360
C	-0.736937777	3.524771422	0.420495993
C	-0.840842767	3.752857331	1.821188633
H	-0.408748041	3.022124125	2.500941839
C	-1.541340812	4.839885658	2.325485894
H	-1.624331913	4.959512187	3.403231193
C	-2.177984086	5.753107946	1.469564485
H	-2.726318779	6.599890553	1.871839770
C	-2.095507568	5.543880681	0.094517532
H	-2.565823638	6.246336279	-0.591019474
C	-1.389994960	4.455049505	-0.433811984
H	-1.275337231	4.358716041	-1.509509880
C	-3.517509348	2.685346236	-4.102830462
H	-3.939144111	3.524862784	-4.672948593
H	-3.744788272	1.745157806	-4.624759231
C	-5.450503281	2.484701633	-2.747582888
H	-5.729234961	1.514039806	-3.178221677
H	-5.956974222	3.290746985	-3.296701759
C	2.539061948	-0.541310156	2.383658555
C	3.664756278	-1.385650886	2.230826112
H	4.165288787	-1.431262409	1.268424515
C	4.163352575	-2.120477426	3.307812946
H	5.032601831	-2.756879236	3.154057740
C	3.576586368	-2.041727908	4.571757737
H	3.974227991	-2.612621645	5.406019265
C	2.472862885	-1.195358287	4.744078419
H	2.003925893	-1.107823469	5.722479484
C	1.964995826	-0.458010823	3.680079431
H	1.109832268	0.198235029	3.823926883
C	3.524824313	0.736891104	-0.420553604

C	3.752897131	0.840930532	-1.821238460
H	3.022142943	0.408925510	-2.501024179
C	4.839952070	1.541434270	-2.325469542
H	4.959578620	1.624525502	-3.403207083
C	5.753200499	2.177962432	-1.469488882
H	6.600001204	2.726305650	-1.871714942
C	5.543976038	2.095364558	-0.094448752
H	6.246449417	2.565593837	0.591129411
C	4.455131580	1.389823168	0.433815094
H	4.358754639	1.275111542	1.509502773
C	2.685230753	3.517354336	4.102955083
H	3.524705979	3.939022020	4.673108743
H	1.745012732	3.744573956	4.624856531
C	2.484641720	5.450379188	2.747744123
H	1.513953783	5.729121188	3.178316759
H	3.290658390	5.956810547	3.296940518
C	-0.541125791	-2.538935686	-2.383807323
C	-1.385561497	-3.664567621	-2.231047271
H	-1.431261059	-4.165113729	-1.268657226
C	-2.120381184	-4.163074894	-3.308078736
H	-2.756856683	-5.032279620	-3.154376109
C	-2.041536500	-3.576273280	-4.572002175
H	-2.612427548	-3.973843116	-5.406299994
C	-1.195077389	-2.472608187	-4.744251052
H	-1.107469047	-2.003642948	-5.722631993
C	-0.457732105	-1.964833173	-3.680204877
H	0.198580615	-1.109713103	-3.823997333
C	0.736934714	-3.524779180	0.420502460
C	0.840839756	-3.752866073	1.821195094
H	0.408747499	-3.022131782	2.500948676
C	1.541335055	-4.839896506	2.325491535
H	1.624326346	-4.959523723	3.403236765
C	2.177975254	-5.753120487	1.469569544
H	2.726307682	-6.599904735	1.871844358
C	2.095498783	-5.543892428	0.094522736
H	2.565812763	-6.246348926	-0.591014770
C	1.389989028	-4.455059051	-0.433806129
H	1.275331802	-4.358724460	-1.509503916
C	3.517533436	-2.685324561	-4.102803130
H	3.939181942	-3.524826419	-4.672932729

H	3.744802735	-1.745124722	-4.624715495
C	5.450517092	-2.484672571	-2.747541824
H	5.729234537	-1.513996996	-3.178158609
H	5.957003856	-3.290698439	-3.296674744
C	-2.539058223	0.541301933	2.383664260
C	-3.664753912	1.385642033	2.230838855
H	-4.165286411	1.431260308	1.268437686
C	-4.163349971	2.120460508	3.307831187
H	-5.032600434	2.756861953	3.154081362
C	-3.576581378	2.041704423	4.571774523
H	-3.974222784	2.612592153	5.406040273
C	-2.472855858	1.195336077	4.744087997
H	-2.003916807	1.107796264	5.722487633
C	-1.964989608	0.457995749	3.680083590
H	-1.109824547	-0.198249399	3.823925428
C	-3.524825594	-0.736889305	-0.420551261
C	-3.752899088	-0.840928537	-1.821235894
H	-3.022145044	-0.408924784	-2.501022915
C	-4.839955377	-1.541430958	-2.325466426
H	-4.959581991	-1.624522210	-3.403203977
C	-5.753204256	-2.177957442	-1.469485315
H	-6.600005755	-2.726299184	-1.871711503
C	-5.543979002	-2.095359985	-0.094445120
H	-6.246452645	-2.565588180	0.591133496
C	-4.455133152	-1.389820660	0.433817977
H	-4.358754587	-1.275110235	1.509505477
C	-2.685220768	-3.517350388	4.102964629
H	-3.524693400	-3.939016754	4.673123074
H	-1.745000270	-3.744570183	4.624861563
C	-2.484640291	-5.450377389	2.747755591
H	-1.513948201	-5.729118965	3.178319093
H	-3.290651887	-5.956806843	3.296961151
H	5.761203881	-2.512840639	-1.700488018
H	2.434049528	-2.804806793	-4.028731332
H	-2.434024323	2.804813323	-4.028751071
H	-5.761194991	2.512852741	-1.700530129
H	2.804759569	2.433877005	4.028857557
H	2.512883073	5.761101962	1.700703096
H	-2.512892540	-5.761102597	1.700715553
H	-2.804749123	-2.433873079	4.028866347

Lowest Energy Frequencies (cm^{-1}) 5.32, 11.99, 14.77, 14.78, 20.94, 22.52

Sum of Electronic and Thermal Free Energies (Hartrees) –4034.95

Table S6. $K_4(OMe_2)_4[Ce(PhNNPh)_4]$ (3^+**-OMe_2**)

Ce	0.000004542	-0.000002717	-0.000005910
K	2.396475588	-2.205177769	1.725300144
O	4.197298814	-4.231754275	1.592422541
N	1.480418943	0.669690245	1.727596604
N	2.306249343	0.690601599	0.550414349
C	1.414352174	1.822119742	2.491599993
C	2.294587420	2.926731020	2.364772606
H	3.082789920	2.890652509	1.621461015
C	2.167074885	4.045915829	3.187537985
H	2.864970976	4.871819150	3.062104191
C	1.170726446	4.125709525	4.163647226
H	1.086830838	4.997743264	4.805984823
C	0.300812061	3.036357093	4.314617256
H	-0.466316249	3.057808262	5.087810359
C	0.415531085	1.909530716	3.503727831
H	-0.249230131	1.061275996	3.643984668
C	3.636757615	0.388654414	0.731359874
C	4.467038460	0.143764131	-0.403331039
H	4.017789323	0.106682475	-1.391476408
C	5.823782107	-0.125494919	-0.266837436
H	6.409869036	-0.319903431	-1.162833783
C	6.434973310	-0.174977870	0.995941296
H	7.499059644	-0.370973714	1.093167328
C	5.637712644	0.042944985	2.122180578
H	6.084896101	0.021173883	3.114827295
C	4.270956789	0.314567827	2.007984895
H	3.689593985	0.530197529	2.897765630
C	5.602899766	-4.029545757	1.656971862
H	6.084663898	-4.334038167	0.716439456
H	6.046015491	-4.603797642	2.484807283
C	3.856757230	-5.579407154	1.291943332
H	4.187027510	-6.257528695	2.093234804

H	4.317208934	-5.896833069	0.345183821
K	2.205174970	2.396463549	-1.725320237
O	4.231753010	4.197285838	-1.592448036
N	-0.669693203	1.480408820	-1.727607182
N	-0.690601081	2.306240929	-0.550426409
C	-1.822127298	1.414338765	-2.491603962
C	-2.926740947	2.294570444	-2.364769357
H	-2.890656060	3.082779289	-1.621464825
C	-4.045937271	2.167044675	-3.187517014
H	-4.871841445	2.864938924	-3.062078664
C	-4.125741048	1.170685953	-4.163614904
H	-4.997783788	1.086780116	-4.805938874
C	-3.036388897	0.300772204	-4.314589300
H	-3.057849876	-0.466367007	-5.087771258
C	-1.909551089	0.415504094	-3.503717491
H	-1.061299274	-0.249260582	-3.643975101
C	-0.388653890	3.636748789	-0.731374378
C	-0.143761718	4.467030946	0.403314897
H	-0.106679465	4.017783094	1.391460596
C	0.125497582	5.823774275	0.266819105
H	0.319907300	6.409862156	1.162814516
C	0.174979355	6.434963838	-0.995960426
H	0.370975257	7.499050066	-1.093188114
C	-0.042945275	5.637701796	-2.122198369
H	-0.021175569	6.084884036	-3.114845705
C	-0.314568738	4.270946205	-2.008000399
H	-0.530200217	3.689582374	-2.897780140
C	4.029544916	5.602886960	-1.656994633
H	4.334042078	6.084649716	-0.716463031
H	4.603793191	6.046003425	-2.484832171
C	5.579407419	3.856743144	-1.291976829
H	6.257524515	4.187013767	-2.093271910
H	5.896839101	4.317193820	-0.345218755
K	-2.396460687	2.205170694	1.725307579
O	-4.197280896	4.231750914	1.592438559
N	-1.480411407	-0.669696277	1.727602419
N	-2.306239622	-0.690606351	0.550419069
C	-1.414357672	-1.822124139	2.491608724
C	-2.294618821	-2.926715907	2.364793588
H	-3.082816094	-2.890629252	1.621476499

C	-2.167145493	-4.045886306	3.187584600
H	-2.865061280	-4.871774276	3.062159866
C	-1.170810241	-4.125685325	4.163706764
H	-1.086944511	-4.997707804	4.806063538
C	-0.300874720	-3.036348441	4.314669423
H	0.466238647	-3.057799752	5.087877506
C	-0.415556654	-1.909535145	3.503756375
H	0.249215028	-1.061288643	3.644012292
C	-3.636747228	-0.388659770	0.731367774
C	-4.467030776	-0.143768146	-0.403320736
H	-4.017784232	-0.106686438	-1.391467274
C	-5.823773905	0.125491336	-0.266822880
H	-6.409863321	0.319900655	-1.162817357
C	-6.434961615	0.174973037	0.995957647
H	-7.499047685	0.370968795	1.093186797
C	-5.637698092	-0.042951700	2.122194543
H	-6.084878903	-0.021181886	3.114842488
C	-4.270942702	-0.314574875	2.007994600
H	-3.689576839	-0.530206562	2.897773176
C	-5.602882145	4.029544805	1.656989436
H	-6.084647017	4.334040247	0.716458401
H	-6.045995594	4.603795684	2.484826762
C	-3.856737370	5.579403979	1.291962192
H	-4.187005300	6.257524039	2.093255850
H	-4.317189941	5.896833069	0.345204176
K	-2.205167927	-2.396469286	-1.725317681
O	-4.231745919	-4.197291702	-1.592445846
N	0.669698892	-1.480410820	-1.727610437
N	0.690610738	-2.306245851	-0.550431854
C	1.822131119	-1.414338283	-2.491609682
C	2.926746049	-2.294568867	-2.364778924
H	2.890663378	-3.082779459	-1.621476081
C	4.045940865	-2.167039658	-3.187528079
H	4.871846154	-2.864933135	-3.062092724
C	4.125741767	-1.170678455	-4.163623699
H	4.997783370	-1.086769903	-4.805948854
C	3.036388474	-0.300765421	-4.314593914
H	3.057847373	0.466375937	-5.087773787
C	1.909552111	-0.415500752	-3.503720608
H	1.061299401	0.249263486	-3.643974990

C	0.388665687	-3.636753673	-0.731383597
C	0.143778567	-4.467040021	0.403303041
H	0.106697757	-4.017795662	1.391449875
C	-0.125478428	-5.823783271	0.266803225
H	-0.319884377	-6.409874380	1.162797301
C	-0.174963451	-6.434968653	-0.995978243
H	-0.370957665	-7.499054882	-1.093209070
C	0.042956536	-5.637702378	-2.122213911
H	0.021185222	-6.084881178	-3.114862766
C	0.314578269	-4.270946766	-2.008011660
H	0.530206853	-3.689579617	-2.897789993
C	-4.029537619	-5.602892728	-1.656995246
H	-4.334032611	-6.084657072	-0.716463762
H	-4.603787656	-6.046007923	-2.484832218
C	-5.579399693	-3.856749870	-1.291970600
H	-6.257518694	-4.187019292	-2.093264544
H	-5.896828835	-4.317202356	-0.345212546
H	2.962545980	5.777334738	-1.814361612
H	5.629562733	2.770168560	-1.191443312
H	-5.629555112	-2.770175503	-1.191434993
H	-2.962538994	-5.777339977	-1.814364866
H	-5.777330823	2.962546514	1.814359532
H	-2.770162993	5.629557600	1.191425881
H	5.777346645	-2.962547467	1.814344053
H	2.770182758	-5.629562309	1.191408672

Lowest Energy Frequencies (cm^{-1}) 13.02, 13.06, 13.06, 13.79, 16.74, 23.47

Sum of Electronic and Thermal Free Energies (Hartrees) –5785.36

Optimized Coordinates for Ce(III) Calculations:

Table S7. $Li_4(OMe_2)_4[Ce(PhNNPh)_4]^-$ (1^--OMe_2)

Ce	-0.000108261	-0.000247107	-0.000935697
O	-5.237741101	0.019168931	0.155101624
O	-0.017817705	5.237653411	-0.151399635
N	-2.042548969	1.441937353	0.589953251
N	-1.442537064	2.043098694	-0.589481352
C	-2.353443508	2.282594406	1.639253986

C	-2.479379772	3.693620113	1.543395988
H	-2.337609403	4.170406613	0.579978731
C	-2.825262452	4.463822745	2.656178896
H	-2.921539146	5.542684105	2.537753372
C	-3.069053633	3.879608152	3.900423746
H	-3.338141460	4.487105031	4.760581182
C	-2.960059021	2.485289322	4.010242599
H	-3.138469681	2.000801409	4.968852170
C	-2.610841720	1.702747615	2.916025600
H	-2.508864999	0.624152225	3.024803540
C	-2.284158215	2.354906509	-1.637724701
C	-1.705407636	2.613634236	-2.914730608
H	-0.626909053	2.511758503	-3.024534173
C	-2.488912725	2.963861366	-4.007927851
H	-2.005251885	3.143254914	-4.966770006
C	-3.883157233	3.072596792	-3.896824674
H	-4.491408321	3.342471797	-4.756202425
C	-4.466297559	2.827474445	-2.652343276
H	-5.545071171	2.923506251	-2.532913099
C	-3.695119214	2.480578729	-1.540541860
H	-4.171103693	2.337816470	-0.576875365
C	-5.902935064	0.372503687	1.362562486
H	-5.193806546	0.942245781	1.966460478
H	-6.210788268	-0.527016636	1.912260699
C	0.722688098	6.049815618	0.749808935
H	0.124760182	6.916328970	1.066285748
H	1.652515888	6.403748042	0.282163204
Li	-3.270917454	0.011776630	0.020278433
Li	-0.011565971	3.270624867	-0.020648180
C	-6.051074213	-0.719873240	-0.746209968
H	-5.436474845	-0.960870254	-1.615912893
H	-6.918281052	-0.121647240	-1.060220811
C	-0.374876784	5.904974090	-1.356544867
H	0.522911408	6.215395180	-1.907667837
H	-0.995127014	6.785874977	-1.136813148
O	0.017794198	-5.238115039	-0.152416692
N	-2.072156278	-1.437237328	-0.573520895
N	-1.438605431	-2.071997540	0.569573026
C	-2.446726635	-2.246291894	-1.626029360
C	-2.608295451	-3.656015069	-1.555110544

H	-2.437813290	-4.154090798	-0.607585828
C	-3.036820303	-4.390414335	-2.663570767
H	-3.167266729	-5.467839021	-2.561958209
C	-3.323895278	-3.771977447	-3.881714706
H	-3.656889428	-4.351596311	-4.738755171
C	-3.175823054	-2.379232210	-3.967606539
H	-3.386928644	-1.867989730	-4.905374571
C	-2.749123075	-1.631715169	-2.876826462
H	-2.621773474	-0.554553648	-2.968985855
C	-2.249093983	-2.449213700	1.619973551
C	-1.636599623	-2.751878231	2.871741873
H	-0.559907059	-2.622367772	2.966387986
C	-2.385601911	-3.181407234	3.960410078
H	-1.875959992	-3.392573859	4.899036202
C	-3.777854982	-3.332166482	3.871344134
H	-4.358645819	-3.667365810	4.726729052
C	-4.394254453	-3.044887510	2.652186513
H	-5.471176436	-3.177498863	2.548070741
C	-3.658361822	-2.613557637	1.545818165
H	-4.154660510	-2.443132876	0.597343380
C	0.374283057	-5.905057383	-1.357955468
H	-0.523774553	-6.214813032	-1.909006180
H	0.994189502	-6.786329435	-1.138747217
Li	0.011325911	-3.271181027	-0.020914158
C	-0.722696094	-6.050438089	0.748660165
H	-0.124847087	-6.917133266	1.064787304
H	-1.652649944	-6.404092166	0.281059104
O	5.237404999	-0.020011490	0.156403940
N	2.042250328	-1.442504054	0.590198905
N	1.442366971	-2.043556650	-0.589349539
C	2.352900414	-2.283228043	1.639501800
C	2.478717284	-3.694264418	1.543608749
H	2.337052957	-4.171003361	0.580151618
C	2.824345150	-4.464544395	2.656416301
H	2.920541297	-5.543409819	2.537959677
C	3.067985298	-3.880410506	3.900729897
H	3.336872493	-4.487971431	4.760904446
C	2.959097813	-2.486086829	4.010591242
H	3.137387963	-2.001656427	4.969252858
C	2.610136184	-1.703467360	2.916348525

H	2.508234357	-0.624866884	3.025156734
C	2.284104445	-2.355318907	-1.637511676
C	1.705499046	-2.613940873	-2.914604198
H	0.627016222	-2.512016694	-3.024528087
C	2.489119993	-2.964130971	-4.007732309
H	2.005569163	-3.143443095	-4.966645507
C	3.883345921	-3.072918902	-3.896468739
H	4.491690468	-3.342762119	-4.755790297
C	4.466347942	-2.827881165	-2.651903255
H	5.545105726	-2.923942245	-2.532355262
C	3.695052781	-2.481029894	-1.540170574
H	4.170930128	-2.338292714	-0.576447594
C	5.901957198	-0.372659743	1.364429476
H	5.192719711	-0.942633086	1.967978475
H	6.208922654	0.527180255	1.914095214
Li	3.270614712	-0.012251544	0.020724643
C	6.051004150	0.719188315	-0.744537001
H	5.436883528	0.959683029	-1.614717133
H	6.918693704	0.121279137	-1.057818531
N	2.072091347	1.436615963	-0.573558017
N	1.438533876	2.071441317	0.569488580
C	2.446250117	2.245520623	-1.626339844
C	2.607278865	3.655321735	-1.555864600
H	2.436673792	4.153632684	-0.608489086
C	3.035407099	4.389553539	-2.664590068
H	3.165434929	5.467061290	-2.563322455
C	3.322599397	3.770859856	-3.882572084
H	3.655283180	4.350349214	-4.739821008
C	3.175053318	2.378032544	-3.968027643
H	3.386265844	1.866584855	-4.905659586
C	2.748755979	1.630682590	-2.876977907
H	2.621824053	0.553443571	-2.968794727
C	2.249081648	2.449155723	1.619649816
C	1.636743787	2.751833415	2.871494414
H	0.560120672	2.621924046	2.966403269
C	2.385823033	3.181856855	3.959916694
H	1.876308280	3.393013172	4.898614326
C	3.778001458	3.333111762	3.870520432
H	4.358851839	3.668693939	4.725714344
C	4.394247425	3.045817592	2.651283938

H	5.471097113	3.178812662	2.546909086
C	3.658279064	2.613998537	1.545158858
H	4.154419227	2.443581841	0.596595568
H	-0.965353327	-5.434750309	1.617127503
H	0.944578061	-5.196445379	-1.961943960
H	-6.403968656	-1.650729491	-0.279845375
H	-6.785164451	0.992030745	1.146142601
H	-0.944985464	5.196386216	-1.960739939
H	0.965575745	5.433896640	1.618047340
H	6.784671152	-0.991763092	1.148773062
H	6.403160867	1.650323808	-0.278177839

Lowest Energy Frequencies (cm^{-1}) 6.79, 14.95, 18.41, 19.81, 20.11, 23.06

Sum of Electronic and Thermal Free Energies (Hartrees) –3416.00

Table S8. $Na_4(OMe_2)_4[Ce(PhNNPh)_4]^-$ (2^--OMe_2)

Ce	-0.000014617	-0.000013032	-0.000018678
Na	-2.580653968	2.159335361	-0.906684583
Na	2.159330551	2.580626080	0.906666353
Na	2.580613771	-2.159373991	-0.906690531
Na	-2.159362265	-2.580643516	0.906680725
O	-4.116090130	2.703950584	-2.571805288
O	2.703966046	4.116045774	2.571795773
O	4.116034312	-2.704027145	-2.571816099
O	-2.703992807	-4.116063491	2.571811024
N	-0.272541848	2.091614326	-1.436148948
N	0.003664597	2.564367061	-0.090681911
N	2.091610622	0.272510949	1.436112837
N	2.564362024	-0.003690754	0.090644291
N	0.272505086	-2.091633858	-1.436146932
N	-0.003692374	-2.564379545	-0.090670484
N	-2.091629085	-0.272532100	1.436127056
N	-2.564382122	0.003664853	0.090654673
C	0.490148392	2.609646462	-2.455723310
C	1.368099923	3.722061332	-2.344188172
H	1.447745825	4.235514873	-1.390528006
C	2.089005138	4.187296988	-3.444994210

H	2.747992339	5.045154217	-3.315719061
C	1.972203109	3.587923012	-4.700672607
H	2.535303467	3.959910360	-5.552771335
C	1.101571462	2.495082821	-4.833192725
H	0.988205354	2.006926133	-5.800627004
C	0.379152121	2.016199128	-3.748151157
H	-0.281082138	1.158588654	-3.860111505
C	-0.688823109	3.675328938	0.325398228
C	-0.779250145	3.950226655	1.725485425
H	-0.352031081	3.228332185	2.418093680
C	-1.457976446	5.058942644	2.208083090
H	-1.527014952	5.206190511	3.284529957
C	-2.095501694	5.961003430	1.337340612
H	-2.628804832	6.825442513	1.723625478
C	-2.036868209	5.705114903	-0.031831512
H	-2.513073635	6.390904805	-0.731786274
C	-1.355964555	4.592958896	-0.541784008
H	-1.273965154	4.456777978	-1.615564234
C	-3.650724386	2.505388362	-3.902553277
H	-4.115454842	3.231663144	-4.585761900
H	-3.877649175	1.487589427	-4.249616303
C	-5.517183583	2.500427622	-2.445627185
H	-5.786124637	1.461705859	-2.677126827
H	-6.069389567	3.184909673	-3.106948264
C	2.609646875	-0.490183184	2.455681897
C	3.722058199	-1.368138204	2.344136847
H	4.235507956	-1.447780057	1.390474299
C	4.187293839	-2.089054167	3.444935927
H	5.045147835	-2.748044072	3.315653088
C	3.587923620	-1.972259752	4.700616827
H	3.959910757	-2.535368640	5.552709950
C	2.495086377	-1.101625708	4.833146501
H	2.006931731	-0.988266580	5.800582606
C	2.016202171	-0.379196285	3.748111834
H	1.158592824	0.281038066	3.860079019
C	3.675312999	0.688812763	-0.325438136
C	3.950182601	0.779283372	-1.725527902
H	3.228289925	0.352057456	-2.418133950
C	5.058863226	1.458065173	-2.208128615
H	5.206089338	1.527135774	-3.284576530

C	5.960918338	2.095599343	-1.337386740
H	6.825331439	2.628942831	-1.723674056
C	5.705053518	2.036927398	0.031788063
H	6.390835694	2.513144947	0.731742135
C	4.592928955	1.355974546	0.541743129
H	4.456762589	1.273955967	1.615523684
C	2.505432369	3.650659594	3.902541196
H	3.231713649	4.115389743	4.585743035
H	1.487637026	3.877568041	4.249625188
C	2.500429612	5.517139396	2.445640631
H	1.461709601	5.786068491	2.677162086
H	3.184918230	6.069341518	3.106958012
C	-0.490196042	-2.609640159	-2.455730338
C	-1.368204679	-3.722013642	-2.344209640
H	-1.447892619	-4.235463146	-1.390552252
C	-2.089124224	-4.187207943	-3.445024569
H	-2.748154913	-5.045033326	-3.315759405
C	-1.972281713	-3.587834956	-4.700699363
H	-2.535392511	-3.959791126	-5.552804832
C	-1.101601847	-2.495031708	-4.833204494
H	-0.988208154	-2.006872453	-5.800634307
C	-0.379172486	-2.016185163	-3.748152982
H	0.281098501	-1.158601778	-3.860103033
C	0.688821272	-3.675312792	0.325453852
C	0.779212144	-3.950170107	1.725551969
H	0.351913122	-3.228289205	2.418125579
C	1.457987056	-5.058832756	2.208205859
H	1.526983720	-5.206053883	3.284659283
C	2.095616017	-5.960867114	1.337512727
H	2.628963268	-6.825259576	1.723841488
C	2.037040197	-5.705003723	-0.031667129
H	2.513342833	-6.390764943	-0.731583774
C	1.356084282	-4.592905708	-0.541676278
H	1.274163892	-4.456733363	-1.615461421
C	3.650714919	-2.505315696	-3.902558384
H	4.115378932	-3.231590594	-4.585812050
H	3.877759953	-1.487516130	-4.249540991
C	5.517148975	-2.500672192	-2.445593831
H	5.786215391	-1.461963770	-2.677002460
H	6.069288494	-3.185165371	-3.106959250

C	-2.609638905	0.490164926	2.455710827
C	-3.722027047	1.368154873	2.344193881
H	-4.235484001	1.447830588	1.390539219
C	-4.187228645	2.089067311	3.445010128
H	-5.045065516	2.748083622	3.315748060
C	-3.587847937	1.972236124	4.700682392
H	-3.959809642	2.535341599	5.552788956
C	-2.495031935	1.101571711	4.833184390
H	-2.006868415	0.988184605	5.800612875
C	-2.016179617	0.379147737	3.748131926
H	-1.158587315	-0.281112193	3.860079305
C	-3.675323688	-0.688843969	-0.325451259
C	-3.950203858	-0.779238672	-1.725544428
H	-3.228325671	-0.351957334	-2.418131590
C	-5.058886885	-1.457995554	-2.208176336
H	-5.206125808	-1.526997378	-3.284626955
C	-5.960921566	-2.095599073	-1.337464577
H	-6.825330909	-2.628931194	-1.723776203
C	-5.705039336	-2.037013802	0.031711217
H	-6.390803256	-2.513291471	0.731642248
C	-4.592920488	-1.356075333	0.541698324
H	-4.456737342	-1.274136756	1.615481386
C	-2.505359787	-3.650711125	3.902553214
H	-3.231629758	-4.115411233	4.585787602
H	-1.487557946	-3.877683799	4.249576657
C	-2.500541030	-5.517166490	2.445620062
H	-1.461823151	-5.786159087	2.677075402
H	-3.185022917	-6.069341200	3.106967500
H	5.782487602	-2.715039938	-1.407280916
H	2.568178101	-2.653006621	-3.896980522
H	-2.568204369	2.653201533	-3.896941961
H	-5.782566238	2.714681341	-1.407301914
H	2.653258266	2.568141386	3.896913587
H	2.714663454	5.782538879	1.407315492
H	-2.714850217	-5.782535916	1.407302914
H	-2.653122897	-2.568184234	3.896951687

Lowest Energy Frequencies (cm^{-1}) 8.48, 10.33, 10.33, 10.39, 18.23, 23.19

Sum of Electronic and Thermal Free Energies (Hartrees) –4035.01

Table S9. $K_4(OMe_2)_4[Ce(PhNNPh)_4]^-$ (3-OMe$_2$)

Ce	0.001222862	0.003654966	0.000014809
K	2.317243334	-2.179369477	1.686015787
O	4.163405376	-4.162599005	1.269282507
N	1.633940348	0.608737127	1.776976932
N	2.445131616	0.602238730	0.577172567
C	1.576730874	1.783769584	2.484634967
C	2.440077682	2.902077811	2.300502600
H	3.213742276	2.847446012	1.542966969
C	2.313083218	4.051058210	3.079292403
H	2.998822736	4.879937550	2.904708880
C	1.334376256	4.165513877	4.071332943
H	1.251781896	5.063374663	4.678343815
C	0.476982591	3.073087191	4.275688427
H	-0.284532721	3.119760677	5.054776146
C	0.588767671	1.915671733	3.511093201
H	-0.074292998	1.073627437	3.691968863
C	3.773463800	0.342902478	0.742065912
C	4.596736936	0.106922694	-0.408305513
H	4.126986699	0.045229637	-1.386533606
C	5.961664320	-0.118340278	-0.295907220
H	6.534834878	-0.301980485	-1.203726050
C	6.603004497	-0.141846303	0.955718459
H	7.674833805	-0.305283173	1.033598142
C	5.815777878	0.050651078	2.095124861
H	6.281119417	0.039195921	3.080836172
C	4.440147900	0.279213119	2.009311838
H	3.867824105	0.476061688	2.909356030
C	5.516079296	-3.821094272	1.001713757
H	5.724806012	-3.868145696	-0.076906889
H	6.203472393	-4.503038314	1.527843866
C	3.828683859	-5.458941170	0.794300454
H	4.402904993	-6.232693560	1.328528440
H	4.030785244	-5.546516618	-0.283163174
K	2.184042097	2.319418903	-1.686770357
O	4.167421249	4.165668440	-1.271396766
N	-0.604215213	1.636039531	-1.777110560
N	-0.597478506	2.447459562	-0.577468166

C	-1.779343328	1.578781267	-2.484600576
C	-2.897563294	2.442243716	-2.300479427
H	-2.842781087	3.216024422	-1.543072174
C	-4.046633891	2.315222448	-3.079130586
H	-4.875442686	3.001051837	-2.904564706
C	-4.161267616	1.336377404	-4.071014785
H	-5.059199417	1.253760542	-4.677917488
C	-3.068921497	0.478881457	-4.275370318
H	-3.115726833	-0.282731681	-5.054354747
C	-1.911415942	0.590692951	-3.510914646
H	-1.069422452	-0.072429683	-3.691810221
C	-0.338069234	3.775736770	-0.742672492
C	-0.101749795	4.599213353	0.407482142
H	-0.039899019	4.129653202	1.385790985
C	0.123645769	5.964092132	0.294764008
H	0.307550848	6.537423507	1.202427915
C	0.146944824	6.605182749	-0.956994051
H	0.310479230	7.676979460	-1.035121521
C	-0.045894470	5.817752291	-2.096202706
H	-0.034610926	6.282898670	-3.082008431
C	-0.274593078	4.442165679	-2.010065683
H	-0.471682829	3.869676802	-2.909951931
C	3.826313774	5.518640143	-1.004858525
H	3.874352178	5.728421880	0.073516698
H	4.507870708	6.205445483	-1.532257109
C	5.464025664	3.831156805	-0.796986557
H	6.237530293	4.404675969	-1.332326832
H	5.552387417	4.034338040	0.280210010
K	-2.314078626	2.186773461	1.687091488
O	-4.160214188	4.170373481	1.271778874
N	-1.630853202	-0.601594161	1.777471724
N	-2.442511284	-0.594975265	0.577996570
C	-1.573529399	-1.776632702	2.485097405
C	-2.437106976	-2.894813948	2.301268669
H	-3.211019749	-2.840057565	1.543993440
C	-2.310021493	-4.043803205	3.080028562
H	-2.995953977	-4.872579361	2.905702908
C	-1.331000333	-4.158393110	4.071744055
H	-1.248338238	-5.056258795	4.678738184
C	-0.473381281	-3.066088859	4.275803301

H	0.288385858	-3.112864355	5.054638809
C	-0.585246505	-1.908667019	3.511230051
H	0.077990415	-1.066714091	3.691889032
C	-3.770724181	-0.335401316	0.743438016
C	-4.594434433	-0.099221973	-0.406576801
H	-4.125090980	-0.037644280	-1.385006253
C	-5.959259791	0.126350063	-0.293594793
H	-6.532776590	0.310133269	-1.201166055
C	-6.600070368	0.149966103	0.958302008
H	-7.671829931	0.313645991	1.036635106
C	-5.812411940	-0.042763808	2.097369520
H	-6.277338287	-0.031258594	3.083276970
C	-4.436871161	-0.271650061	2.010966089
H	-3.864217709	-0.468687474	2.910759519
C	-5.513158608	3.829132527	1.005259017
H	-5.722817681	3.876614596	-0.073163303
H	-6.200020040	4.510961881	1.532232497
C	-3.825681598	5.466774264	0.796830381
H	-4.399223999	6.240505328	1.331818362
H	-4.028812916	5.554681982	-0.280412914
K	-2.182109271	-2.311910884	-1.686265352
O	-4.165566965	-4.157949516	-1.269627795
N	0.606108360	-1.628758634	-1.777285384
N	0.599731716	-2.440184899	-0.577642122
C	1.781032298	-1.571476599	-2.485109428
C	2.899316929	-2.434933291	-2.301330392
H	2.844770344	-3.208711711	-1.543905232
C	4.048153339	-2.307898064	-3.080325050
H	4.877015782	-2.993732506	-2.906024447
C	4.162482692	-1.329050902	-4.072242000
H	5.060227365	-1.246432109	-4.679420907
C	3.070074437	-0.471552334	-4.276253128
H	3.116646591	0.290076268	-5.055236382
C	1.912805133	-0.583365560	-3.511441892
H	1.070763615	0.079770874	-3.692064147
C	0.340220056	-3.768451624	-0.742763564
C	0.104373073	-4.591947421	0.407475272
H	0.042952075	-4.122403971	1.385820217
C	-0.121076460	-5.956820919	0.294824195
H	-0.304600301	-6.530169069	1.202554351

C	-0.144901076	-6.597891057	-0.956934958
H	-0.308490856	-7.669682794	-1.035011431
C	0.047493939	-5.810447264	-2.096206807
H	0.035814016	-6.275576973	-3.082015909
C	0.276251459	-4.434863822	-2.010138884
H	0.473048197	-3.862382543	-2.910094555
C	-3.823943595	-5.510609614	-1.002127341
H	-3.870474637	-5.719262033	0.076529003
H	-4.506145722	-6.198032186	-1.527884237
C	-5.461713107	-3.823216357	-0.794122248
H	-6.235685211	-4.397426400	-1.328043666
H	-5.548861509	-4.025319769	0.283375787
H	2.802674807	5.682487019	-1.348400856
H	5.612461206	2.762137051	-0.963031371
H	-5.610543467	-2.754393717	-0.961061832
H	-2.800762710	-5.674720708	-1.346903617
H	-5.677053798	2.805672204	1.349308739
H	-2.756669354	5.615279551	0.962851477
H	5.680152289	-2.797744738	1.346008355
H	2.759863531	-5.607717608	0.961311614

Lowest Energy Frequencies (cm^{-1}) 15.11, 16.31, 17.61, 17.61, 20.16, 26.73

Sum of Electronic and Thermal Free Energies (Hartrees) –5785.43

References

1 L. Yang, D. R. Powell and R. P. Houser, *Dalton Trans.*, 2007, 955.
2 E. L. Muetterties and L. J. Guggenberger, *J. Am. Chem. Soc.*, 1974, **96**, 1748; J. L. Hoard and J. V. Silverton, *Inorg. Chem.*, 1963, **2**, 235; S. R. Sofen, S. R. Cooper and K. N. Raymond, *Inorg. Chem.*, 1979, **18**, 1611; W. L. Smith and K. N. Raymond, *J. Am. Chem. Soc.*, 1981, **103**, 3341; D. G. Blight and D. L. Kepert, *Inorg. Chem.*, 1972, **11**, 1556; E. L. Muetterties and C. M. Wright, *Q. Rev. Chem. Soc.*, 1967, **21**, 109; S. J. Lippard and B. J. Russ, *Inorg. Chem.*, 1968, **7**, 1686.
3 M. D. Walter, R. Fandos and R. A. Andersen, *New J. Chem.*, 2006, **30**, 1065.
4 U. J. Williams, D. Schneider, W. L. Dorfner, C. Maichle-Mossmer, P. J. Carroll, R. Anwander and E. J. Schelter, *Dalton Trans.*, 2014, **43**, 16197.
5 M. P. Andersson and P. Uvdal, *J. Phys. Chem. A*, 2005, **109**, 2937; K. K. Irikura, R. D. Johnson III and R. N. Kacker, *J. Phys. Chem. A*, 2005, **109**, 8430.
6 C. A. Smith, E. W. Ainscough, H. M. Baker, A. M. Brodie and E. N. Baker, *J. Am. Chem. Soc.*, 1994, **116**, 7889; J. Robinson, P. J. Carroll, P. J. Walsh and E. J. Schelter, *Angew. Chem. Int. Ed.*, 2012, **51**, 10159.
